# Cerebral blood flow monitoring using a deep learning implementation of the two-layer diffuse correlation spectroscopy analytical model with a 512 × 512 SPAD array

**DOI:** 10.1117/1.NPh.12.3.035008

**Published:** 2025-08-18

**Authors:** Mingliang Pan, Chenxu Li, Yuanzhe Zhang, Alan Mollins, Quan Wang, Ahmet T. Erdogan, Yuanyuan Hua, Zhenya Zang, Neil Finlayson, Robert K. Henderson, David Day-Uei Li

**Affiliations:** a University of Strathclyde, Department of Biomedical Engineering, Glasgow, United Kingdom; b University of Edinburgh, School of Engineering, Integrated Nano and Micro Systems (IMNS), Edinburgh, United Kingdom

**Keywords:** diffuse correlation spectroscopy, deep learning, SPAD, cerebral blood flow, physiological response monitoring

## Abstract

**Significance:**

Multilayer (two- and three-layer) diffuse correlation spectroscopy (DCS) models improve cerebral blood flow index (CBFi) measurement sensitivity and mitigate interference from extracerebral tissues. However, their reliance on multiple predefined parameters (e.g., layer thickness and optical properties) and high computational load limit their feasibility for real-time bedside monitoring.

**Aim:**

We aim to develop a fast, accurate DCS data processing method based on the two-layer DCS analytical model, enabling real-time cerebral perfusion monitoring with enhanced brain sensitivity.

**Approach:**

We employed deep learning (DL) to accelerate DCS data processing. Unlike previous DCS networks trained on single-layer models, our network learns from the two-layer DCS analytical model, capturing extracerebral versus cerebral dynamics. Realistic noise was estimated from subject-specific baseline measurements using a 512×512 SPAD array at a large source-detector separation (35 mm). The model was evaluated on test datasets simulated with a four-layer slab head model via Monte Carlo (MC) methods and compared against conventional single-exponential fitting and the two-layer analytical fitting. Two *in vivo* physiological response tests were also conducted to assess the real-world performance.

**Results:**

The proposed method bypasses traditional curve-fitting and achieves real-time monitoring of CBF changes at 35 mm separation for the first time with a DL approach. Validation on MC simulations shows superior accuracy in relative CBFi estimation (4.1% error versus 12.7% for single-exponential fitting) and significantly enhanced CBFi sensitivity (86.5% versus 57.7%). Although the two-layer analytical fitting offers optimal performance, it depends on strict assumptions and preconditions, and its computational complexity makes it time-consuming and unsuitable for real-time monitoring applications.

In addition, our method minimizes the influence of superficial blood flow and is 750-fold faster than single-exponential fitting in a realistic scenario. *In vivo* tests further validated the method’s ability to support real-time cerebral perfusion monitoring and pulsatile waveform recovery.

**Conclusions:**

This study demonstrates that integrating DL with the two-layer DCS analytical model enables accurate, real-time cerebral perfusion monitoring without sacrificing depth sensitivity. The proposed method enhances CBFi sensitivity and recovery accuracy, supporting future deployment in bedside neuro-monitoring applications.

## 1 Introduction

Cerebral blood flow (CBF) is a critical biomarker for brain health and function, supporting cognitive and neurological processes.^1^ Existing technique, such as transcranial Doppler ultrasound, is noninvasive but relies on experienced operators and is limited to larger arteries.^2^ Functional magnetic resonance imaging (fMRI) and positron emission tomography (PET), on the other hand, are bulky and provide only “snapshot” observations; they are not suitable for bedside applications.^3^^,^^4^ By contrast, diffuse correlation spectroscopy (DCS) offers noninvasive, continuous, high temporal resolution CBF index (CBFi) measurements at the bedside.^5^ DCS analyzes light intensity fluctuations caused by red blood cell movement using the autocorrelation function (ACF), given by $g_{2}=\langle I(t)I(t+\tau )\rangle /{\left\langle I(t)\right\rangle }^{2}$, to assess blood flow dynamics.^6^^,^^7^

Over the past two decades, DCS has evolved from continuous-wave to time-domain (TD-DCS) and frequency-domain variants.^8^^,^^9^ Analytical models for complex tissue structures have advanced from semi-infinite homogeneous medium to two-layer and three-layer models.^10^^,^^11^ These developments have expanded DCS applications in brain health evaluation, neurovascular studies, cancer diagnosis, and therapy evaluation.^3^

Traditionally, DCS maps measured $g_{2}$ curves to the semi-infinite model, allowing real-time CBFi measurements.^12^ However, this model underestimates CBFi and is susceptible to extracerebral layer interference.^13^ Attempts to improve CBFi sensitivity by fitting $g_{2}$ at short correlation times with the semi-infinite analytical model or increasing the source-detector separation (ρ) often reduce signal-to-noise ratio (SNR) and lead to inaccurate CBFi estimates.^14^ There is a trade-off between the detection depth and SNR.^15^ Although advanced DCS variants such as pathlength-resolved DCS or TD-DCS can mitigate this issue, their complexity limits widespread adoption.^16^^,^^17^ Multichannel DCS (MDCS) improves SNR by an order of magnitude, enabling deeper blood flow measurement.^18^^,^^19^ For example, the ATLAS SPAD sensor developed by the University of Edinburgh achieves ρ∼5  cm.^20^ In addition, the two-layer and three-layer analytical models enhance CBFi sensitivity and enable separation of cerebral and extracerebral blood flow but require multiple predefined parameters for fitting (e.g., layer thickness and optical properties). If these parameters are mis-specified, significant errors may occur.^21^^–^^23^ In addition, these models are computationally intensive, limiting real-time applications.^24^ Integrating MDCS with multilayer analytical models could enable more accurate CBFi estimation. However, advanced data processing techniques are required to overcome the existing limitations.

Deep learning (DL) has emerged as an efficient technique for DCS data processing in multiple studies.^25^^–^^29^ It has demonstrated improved speed and robustness compared with traditional curve-fitting approaches.^24^^,^^25^ For example, Poon et al.^25^ achieved 23-fold speed improvement, and Li et al.^27^ showed that long short-term memory (LSTM) could improve BFi accuracy. Nakabayashi et al.^30^ explored an LSTM to separate shallow versus deep flow in a two-layer flow phantom, underlining the community’s interest in accounting for layers. However, to date, these DL models have been trained mostly on the semi-infinite homogeneous (single-layer) analytical data, Monte Carlo simulations, or phantom experimental data, which are not suitable for CBFi recovery. To our knowledge, no prior publication has utilized training data from the two-layer DCS analytical model. The two-layer analytical model can accurately recover CBFi and relative CBFi (rCBFi) compared with the semi-infinite model while requiring fewer parameters than the three-layer model,^22^ making it an ideal candidate for training dataset generation.

To overcome the limitations of traditional fitting and leverage the capabilities of the new ATLAS SPAD sensor,^20^ this paper presents the following innovations.

We incorporated an SPAD-DCS system with a DL model trained on datasets generated by the two-layer DCS analytical model for real-time CBFi estimation. We validated our method using simulated test datasets to assess CBFi waveform recovery, rCBFi estimation, and CBFi sensitivity to brain and scalp blood flow perturbations, comparing the results with traditional single-exponential and two-layer analytical fittings. Finally, we evaluated the capability of the DL-based DCS system to monitor breath-holding and digestion-induced physiological responses in a healthy adult at a large ρ of 35 mm.

## 2 Methods

### 2.1 Theory of the Two-layer Analytical Model

The DCS theory is based on the correlation diffusion equation (CDE), derived from the correlation transfer equation (CTE) under the standard diffusion assumption.^6^ This derivation is analogous to the photon diffusion equation from the radiative transfer equation (RTE) using the $P_{N}$ approximation.^6^^,^^31^ The analogy between the CTE and RTE was first established by Ackerson et al..^32^ The CDE is expressed as

$$(\frac{D}{\upsilon }\nabla^{2}-\mu_{a}-\frac{1}{3}\mu_{s}^{\prime }k_{0}^{2}\alpha \langle \Delta r^{2}(\tau )\rangle )G_{1}(r,\tau )=-S(r),$$ (1)

where $G_{1}(r,\tau )=\langle E(r,t)E^{*}(r,t+\tau )\rangle$ is the unnormalized electric field temporal ACF, $D=\upsilon /(3\mu_{s}^{\prime })$ is the photon diffusion coefficient, υ is the speed of light in the medium, τ is the time lag, $k_{0}=2\pi n_{0}/\lambda$ is the wavenumber of light in the scattering medium at the wavelength λ and $n_{0}$ is the tissue refractive index, $\mu_{a}$ is the absorption coefficient, $\mu_{s}^{\prime }$ is the reduced scattering coefficient, S(r) is the source, and $\left\langle \Delta r^{2}(\tau )\right\rangle$ is the mean square displacement of scatterers. For diffusive motions, $\langle \Delta r^{2}(\tau )\rangle =6D_{b}\tau$, where $D_{b}$ is the effective Brownian diffusion coefficient. In most practical applications, the Brownian motion model is accurate to describe the scatterers’ motions.^23^^,^^33^^,^^34^ The product $\alpha D_{b}$ is defined as BFi,^4^^,^^7^ where α is defined as the ratio of moving scatterers to total scatterers, assumed to be 1 in our simulations.^35^

Following the analytical derivation process developed by Gagnon et al.,^10^ we assume an isotropic source incident at depth $z_{0}=1/(\mu_{a,1}+\mu_{s,1}^{\prime })$ and scatters in each layer present independent Brownian diffusion motion. Then the CDE will be

$$[D_{1}\nabla^{2}-\mu_{a,1}-2\mu_{s,1}^{\prime }k_{0}^{2}D_{b,1}\tau ]G_{1,1}(x,y,z,\tau )=-\delta (x,y,z-z_{0})\;0\leq z\leq l,\;[D_{2}\nabla^{2}-\mu_{a,2}-2\mu_{s,2}^{\prime }k_{0}^{2}D_{b,2}\tau ]G_{1,2}(x,y,z,\tau )=0\;l\leq z,$$ (2)

where j=1,2 refers to the layer indices, $G_{1,j}$, $D_{j}$, $\mu_{a,j}$, $\mu_{s,j}^{\prime }$, and $D_{b,j}$ are the unnormalized electric field temporal ACF, diffusion coefficient, absorption coefficient, reduced scattering coefficient, and Brownian diffusion coefficient in layer j, respectively, l is the thickness of layer 1. The Fourier domain solution to Eq. (2) at the surface of layer 1 is

$${\overset{˜}{G}}_{1,1}(s,z,\tau )=\frac{\sinh[\alpha_{1}(z_{b}+z_{0})]}{D_{1}\alpha_{1}}\times \frac{D_{1}\alpha_{1}\;\cosh[\alpha_{1}(l-z)]+D_{2}\alpha_{2}\;\sinh[\alpha_{1}(l-z)]}{D_{1}\alpha_{1}\;\cosh[\alpha_{1}(l+z_{b})]+D_{2}\alpha_{2}\;\sinh[\alpha_{1}(l+z_{b})]}\;-\frac{\sinh[\alpha_{1}(z_{0}-z)]}{D_{1}\alpha_{1}},$$ (3)

where $\alpha_{j}^{2}=(D_{j}s^{2}+\mu_{a,j}+2v\mu_{s,j}^{\prime }k_{0}^{2}D_{b,j})/D_{j}$ and v is the light speed, $z_{b}=2D_{1}(1+R_{\mathrm{eff}})/(1-R_{\mathrm{eff}})$. The Fourier inversion of Eq. (3) is

$$G_{1}^{1}(\rho ,z,\tau )=\frac{1}{2\pi }\int_{0}^{\infty }{\overset{˜}{G}}_{1,1}(s,z,\tau )sJ_{0}(s\rho )ds,$$ (4)

where $J_{0}$ is the zeroth order Bessel function of the first kind.^10^^,^^36^

The normalized electric field temporal ACF, $g_{1}(\tau )$, is related to the normalized light intensity ACF, $g_{2}(\tau )$, through the Siegert equation ^37^

$$g_{2}(\tau )=1+\beta |g_{1}(\tau ){|}^{2},$$ (5)

where β depends on the laser stability, coherence length, and the number of speckles detected.^34^ The experimentally measured light intensity ACF can be calculated as

$$g_{2}(\tau )=\frac{\left\langle I(t)I(t+\tau )\right\rangle }{{\left\langle I(t)\right\rangle }^{2}},$$ (6)

where ⟨…⟩ denotes the average over the integration time $T_{\mathrm{int}}$ and I(t) is the measured light intensity fluctuation. By fitting the measured light intensity ACF to the analytical solution, the BFi ($\alpha D_{b}$) can be extracted.

### 2.2 Training Dataset Preparation

The clean training dataset was generated using the two-layer DCS analytical model. Based on previous studies,^10^^,^^38^ we varied only the dominant parameters of the model. Specifically, we varied the brain $\mu_{a}$ and $\mu_{s}^{\prime }$, the extracerebral layer thickness, and the extracerebral layer $D_{b}$ ($D_{b\_\text{extra}}$) and brain $D_{b}$ ($D_{b\_\text{brain}}$) while keeping other parameters constant. The brain $\mu_{a}$ varied linearly from 0.005 to $0.025\;{\mathrm{mm}}^{-1}$ with a step size of $0.005\;{\mathrm{mm}}^{-1}$, and the brain $\mu_{s}^{\prime }$ varied from 0.9 to $1.3\;{\mathrm{mm}}^{-1}$ (the step size: $0.1\;{\mathrm{mm}}^{-1}$). The extracerebral layer thickness varied from 8 to 15 mm (the step size: 1 mm). $D_{b\_\text{brain}}$ varied linearly from $5\times 10^{-7}$ to $5\times 10^{-5}\;{\mathrm{mm}}^{2}/s$ with a step of $5\times 10^{-7}\;{\mathrm{mm}}^{2}/s$ (100 steps). Each $D_{b\_\text{brain}}$ corresponded to 20 steps of $D_{b\_\text{extra}}$, with the extracerebral-to-brain $D_{b}$ fraction ranging from 0.05 to 0.3. This relationship was estimated from previous reports.^39^^,^^40^ In total, we simulated 400,000 clean $g_{2}$ data (5×5×8×100×20). The $D_{b}$, physiological and optical properties for the two-layer analytical model were adopted from previous studies^10^^,^^14^^,^^38^^,^^39^^,^^41^^–^^43^ to ensure a thorough coverage of relevant ranges. ρ was fixed at 35 mm, β was assumed to be 0.5, and the time lag τ ranged from 1.28 to 39.68  μs (step size: 1.28  μs) to align with the hardware settings (see Sec. 2.3). We fixed β as a constant because scaling the $g_{2}$ curve to the same range will eliminate the influence of β variance at a given flow rate. As a result, it is unnecessary to vary β to account for potential hardware instability (see Sec. 2.3 for details). The parameter configurations are summarized in Table 1.

**Table 1:** Simulation parameters for the two-layer analytical model.

Tissue type	$\mu_{a}$ (${\mathrm{mm}}^{-1}$)	$\mu_{s}^{\prime }$ (${\mathrm{mm}}^{-1}$)	Tissue thickness (mm)	$D_{b}$ (${\mathrm{mm}}^{2}/s$)
Extracerebral layer	0.019	0.86	(8, 15)	(0.05, 0.3) × $D_{b\_\text{brain}}$
Brain	(0.005, 0.025)	(0.9, 1.3)	∞	($5\times 10^{-7}$, $5\times 10^{-5}$)

### 2.3 SPAD-DCS System and Noise Calculation

**Fig. 1:**
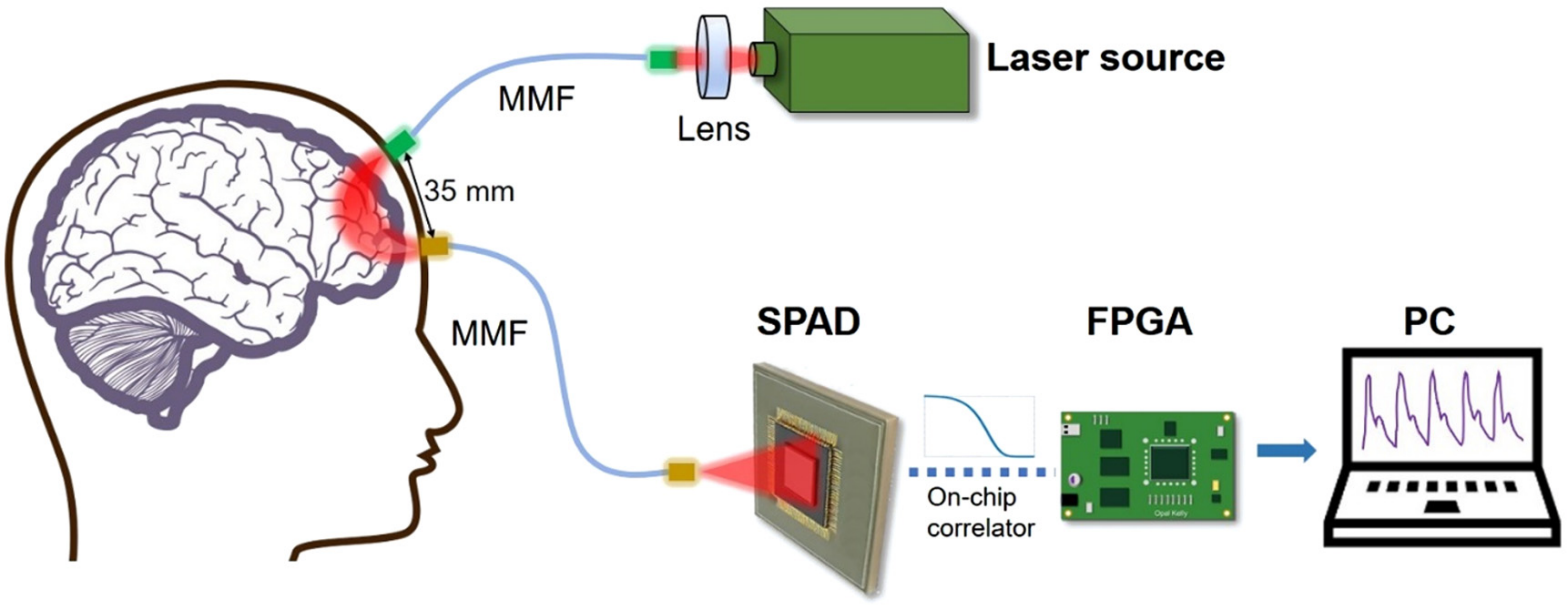
SPAD-DCS system setup. The 785 nm continuous-wave laser was coupled into a multimode fiber (MMF, M143L01, Ø600  μm, 0.22 NA, Thorlabs, Newton, New Jersey, United States), and the scattered photons was collected by another MMF (M59L01, Ø1000  μm, 0.50 NA, Thorlabs, Newton, New Jersey, United States). The output of the SPAD array was received by an Opal Kelly FPGA board (XEM7310-A200) and transferred to the PC through a USB 3.0 cable. The on-chip computed ACF is sent to the trained DL model for real-time CBFi/rCBFi display.

As reported earlier,^20^ the SPAD sensor ATLAS, with embedded on-chip autocorrelation computation optimized for DCS applications, has demonstrated deep and high-speed CBF monitoring. In this work, we operated ATLAS in the ensemble DCS mode, where all 128×128  macropixels (each composed of 4×4  micropixels) were combined and averaged to output 31 time lags of the light intensity ACF. The pixel clock (*PixClk*) was set to 25 MHz, corresponding to a time lag range of 1.28 to 39.68  μs, enabling deeper/faster flow information capture. The iteration number was set to 4096, corresponding to an integration time of 5.24  ms. We used a continuous-wave laser source (DL785-100-S, 785 nm, 100 mW, CrystaLaser, Reno, Nevada, United States) coupled with a multimode optical fiber (MMF, M143L01, Ø600  μm, 0.22 NA, Thorlabs, Newton, New Jersey, United States) to illuminate tissues. The detector fiber (MMF, M59L01, Ø1000  μm, 0.50 NA, Thorlabs, Newton, New Jersey, United States) tip was placed 23 mm from the SPAD chip, the optimal distance for maximizing speckle contrast.^20^ Both fibers were held by a custom 3D-printed probe, maintaining a 35 mm separation. The overall system setup is illustrated in Fig. 1. We processed SPAD ACF data using single-exponential fitting, a simplified model commonly preferred for real-time measurements. The analytical normalized light intensity ACF can be simplified to a single-exponential decay function at a small τ range^44^^,^^45^

$$g_{2}(\tau )=1+\beta e^{-2\tau /\tau_{c}},$$ (7)

where $\tau_{c}$ is the decorrelation time. The reciprocal of $\tau_{c}$, known as the decorrelation speed, is directly proportional to blood flow rate and can be used to quantify blood flow changes.^13^ We simplified Eq. (7) to the following format to facilitate data analysis:

$$g_{2}(\tau )=a+be^{-c\tau }.$$ (8)

The validity of this simplification is demonstrated in Fig. S3 of the Supplementary Material. In the following sections, we compare cerebral perfusion measured by the DL and the single-exponential fitting method through comparing rCBFi measured with the DL model and the relative change in single-exponential fitting-recovered decorrelation speed.

**Fig. 2:**
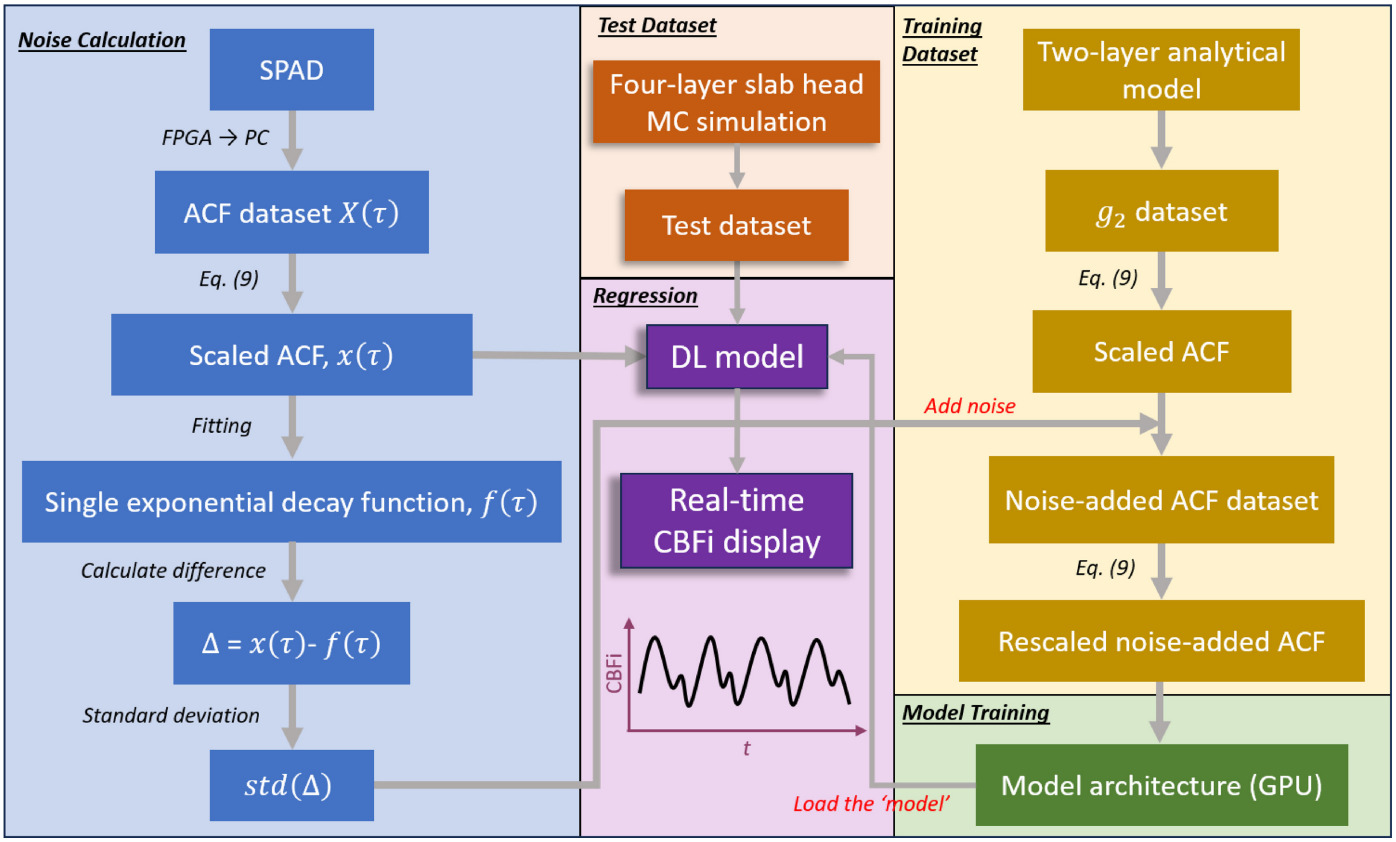
Flowchart of the data processing protocol. The process consists of four main components: (1) the “noise calculation” block, which calculates the standard deviation from experimental baseline data; (2) the “dataset generation” block, which outlines the procedure for generating clean data and adding noise; (3) the “model training” block, which describes the training of the model on a GPU; and (4) the “regression” block, which represents real-time CBFi/rCBFi display using the trained model and the preparation of experimental data before input to the model.

**Fig. 3:**
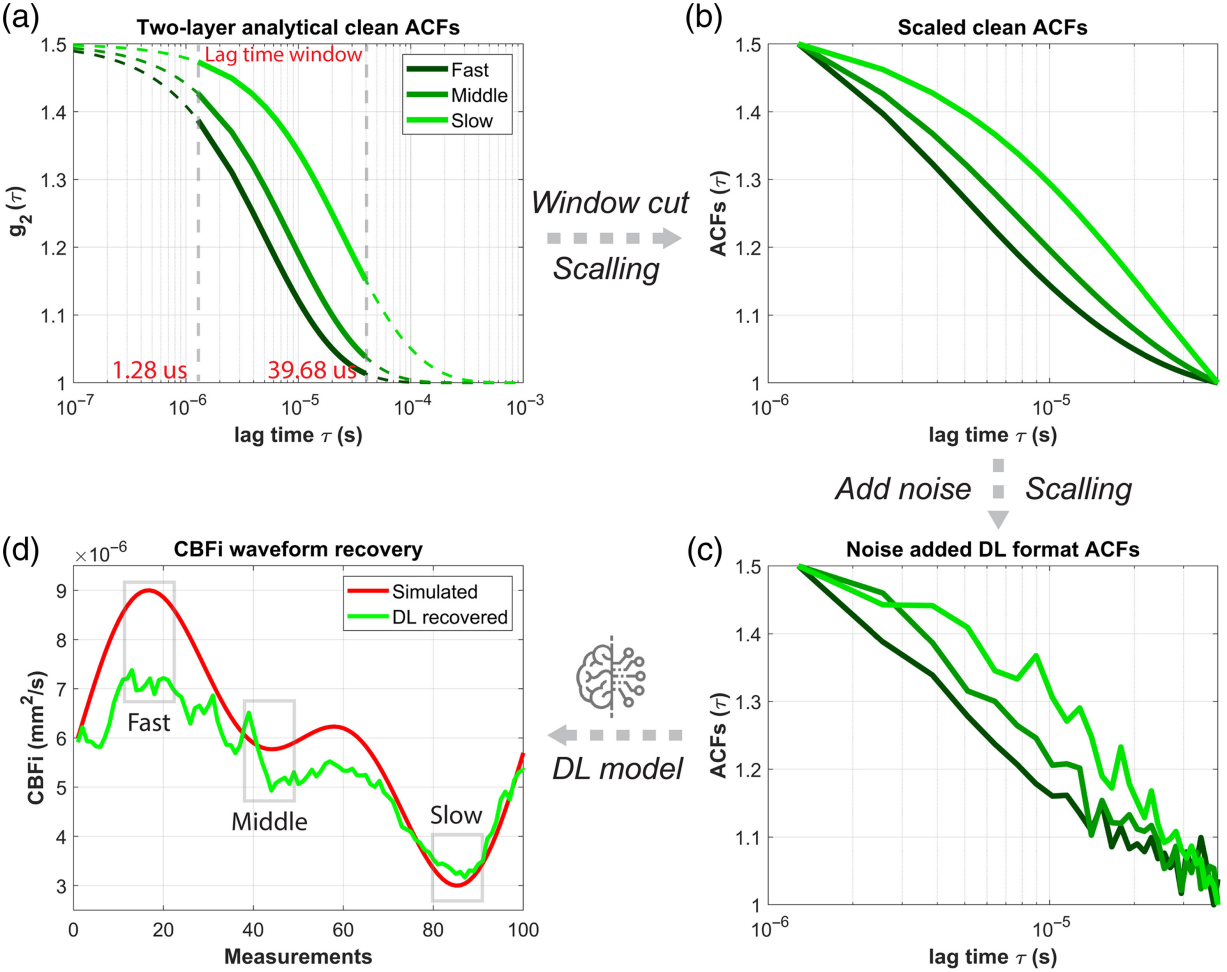
ACF data processing workflow for training and testing the DL model. (a) ACFs were generated using the two-layer analytical model at different flow rates (fast, medium, and slow, as the representatives). Each curve was truncated according to the time lag window used in the SPAD-DCS system. (b) The windowed ACFs were scaled to [1, 1.5]. (c) Experimentally derived noise was added to the scaled ACFs, and the noisy ACFs were rescaled to [1, 1.5]. These processed ACFs were then formatted for input into the DL model. (d) Representative CBFi waveforms recovered by the DL model from the test dataset.

We applied this system to a healthy human adult (male, 29 years old) at the resting state to collect the baseline autocorrelation. The probe was secured to the participant’s forehead using an adjustable Velcro strap to ensure stability and comfort. The test was repeated 5 times, recording 1000 ACF frames per session, yielding a total of 5000 baseline samples for noise estimation. We use X(τ) to represent the collected ACF data. To prevent overestimation of noise due to pulsatile CBF fluctuations, we applied a multistep correction process, as illustrated in Fig. 2 (block with light blue background). First, X(τ) was scaled to [1, 1.5] using the equation

$$x(\tau )=\frac{X(\tau )-\min(X(\tau ))}{\max(X(\tau ))-\min(X(\tau ))}\times 0.5+1,$$ (9)

where x(τ) is the scaled measured ACF. Second, x(τ) was fitted with a single-exponential decay function, $f(\tau )=a+be^{c\tau }$. Third, we calculated the standard deviation of the residual (as a function of τ), i.e., σ=std(x(τ)−f(τ)), *std()* is the standard deviation calculation function used in MATLAB. We obtained five sequences of σ from the five tests and calculated their mean and standard deviation. The averaged sequence was then scaled by ±30% to encompass the variability observed across the tests (actual range is from −27.8% to +30.1% relative to the averaged sequence). This yielded three levels of σ: the averaged σ, +30% and −30% from mean. Finally, the three levels of σ are substituted into a Gaussian distribution model with zero mean to generate noise and added to the simulated clean dataset.^44^ In total, we generated 1,200,000 training data samples (through a combination of 400k clean curves × 3 noise levels). Notably, we rescaled each noise-added curve with the same method [Eq. (9)] prior to training. Likewise, any experimental SPAD ACF is scaled with Eq. (9) before input to the model to maintain consistency between training and inference. The workflow of our method is illustrated in Fig. 2, including five parts: noise calculation, training dataset generation, test dataset generation, model training, and regression. The detailed ACF data processing for training and testing our model is shown in Fig. 3.

### 2.4 Deep Learning Model Architecture

**Fig. 4:**
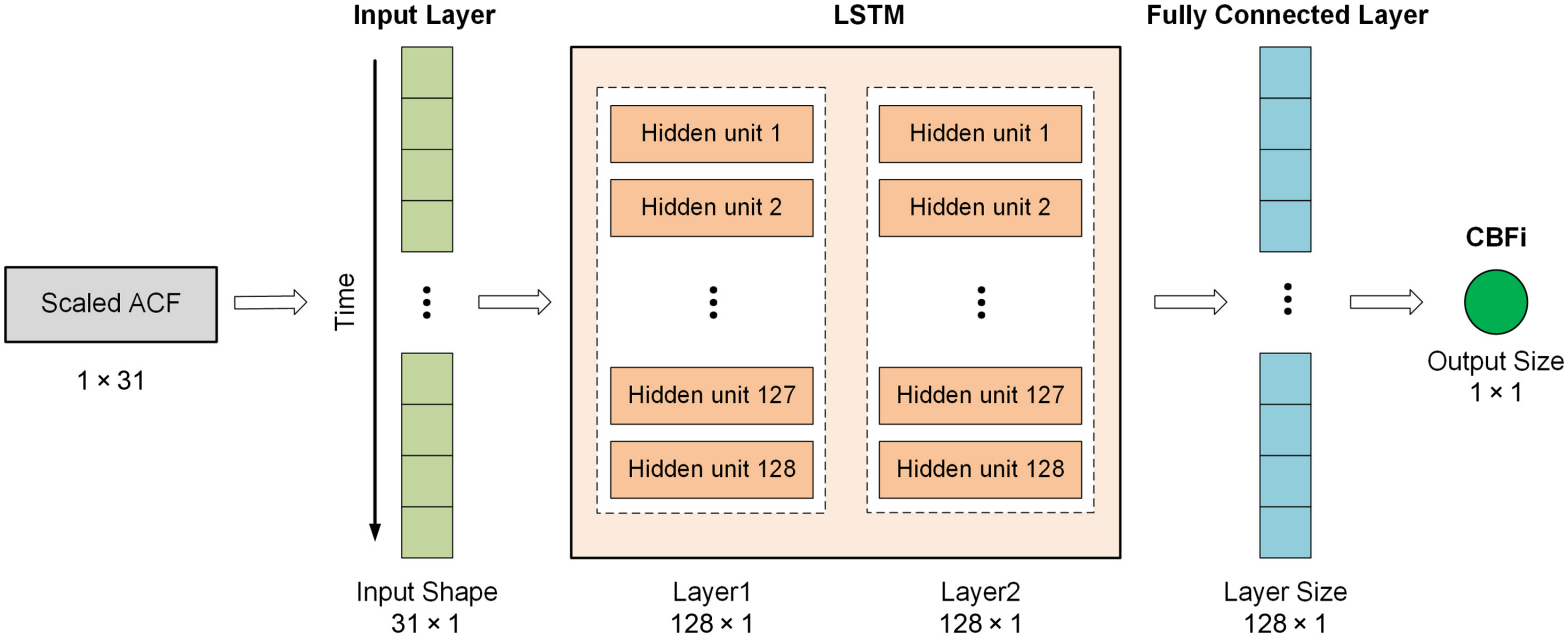
Proposed LSTM model architecture.

In this study, we employed an LSTM network as our deep learning model architecture. LSTM, an advanced variant of the recurrent neural network (RNN) architecture designed for modeling sequential data, has been applied in several studies for DCS data analysis.^30^^,^^46^ Our LSTM model architecture is illustrated in Fig. 4. The dataset consists of 1,200,000 samples, with 80% used for training (960,000 samples) and 20% for validation. CBFi was used as the training label, and each value was scaled by $10^{6}$ to prevent slow training convergence. We chose an LSTM with 2×128 units as it offered a good balance of complexity and performance, and similar RNN-based models have proven effective in DCS analysis.^27^^,^^28^^,^^30^ Based on our model architecture (Table 2), the total number of trainable parameters is 198,273, yielding a training sample-to-parameter ratio of 4.84:1. This ratio supports effective generalization while reducing the risk of overfitting, which is generally sufficient to avoid overfitting. The model was trained by minimizing the mean squared error (MSE) loss function, with Adam as the optimizer. To avoid overfitting, we applied a dropout rate of 0.3 and L2 regularization (weight decay of $10^{-4}$, batch size of 256, and epoch of 1000) during the training. A fully connected layer processes the unit’s output to regress CBFi. The model was developed using PyTorch and executed on our workstation [graphics processing unit (GPU): NVIDIA Quadro RTX 5000 with 16 GB memory]. The training and validation loss curves are shown in Fig. S2 (the Supplementary Material).

**Table 2:** LSTM architecture parameters.

Parameters	Values
Input size	31 × 1
Number of hidden layers	2
Layer unit	128
Loss function	MSE
Optimizer	Adam
Learning rate	0.0001
Batch size	256
Epoch	1,000
Output size	1 × 1

### 2.5 Monte Carlo Simulation for Test Dataset Generation

We conducted Monte Carlo (MC) simulations using the voxel-based Monte Carlo eXtreme (MCX)^47^ toolkit in MATLAB (R2023b, MathWorks, Natick, Massachusetts, United States) to generate the test dataset. We simulated a slab human head model with a volume of $200\times 200\times 100\;{\mathrm{mm}}^{3}$, segmented into four layers, each layer representing scalp, skull, cerebrospinal fluid (CSF), and brain tissues. Light with a wavelength of 785 nm was used in all MC simulations; the layer thicknesses and optical parameters at 785 nm are listed in Table 3. Each simulation was executed with $5\times 10^{8}\;\text{photons}$, and the detector was positioned at ρ=35  mm to record the photon transfer and photon pathlength, thereby enabling the calculation of the temporal light field ACF^7^^,^^48^

$$G_{1}(\tau )=\frac{1}{N_{p}}\overset{N_{p}}{\underset{n=1}{\sum }}\;\exp(\overset{N_{\text{tissue}}}{\underset{i=1}{\sum }}-\frac{1}{3}Y_{n,i}k_{0}^{2}{\left\langle \Delta r^{2}(\tau )\right\rangle }_{i})\exp(-\overset{N_{\text{tissue}}}{\underset{i=1}{\sum }}\mu_{a,i}L_{n,i}),$$ (10)

where $N_{p}$ is the number of detected photons at each detector, $N_{\text{tissue}}$ is the number of tissue types ($N_{\text{tissue}}=4$ in our case), $Y_{n,i}$ and $L_{n,i}$ are the total momentum transfer and total pathlength of photon n in layer i, and $\mu_{a,i}$ is the absorption coefficient in layer i. ${\left\langle \Delta r^{2}(\tau )\right\rangle }_{i}=6D_{bi}\tau$ is the mean square displacement of the scattered particles in layer i, where $D_{bi}$ is the effective Brownian diffusion coefficient in layer i. The correlation delay time τ was set between 1.28 and 39.68  μs with 31 linearly spaced data points to agree with the SPAD output data format. β was set to 0.5, and $g_{2}(\tau )$ curves were obtained by substituting the normalized electric field ACF into Eq. (5). The anisotropic factor g was set to 0.89 for all MC simulations.

**Table 3:** Baseline flow and physiological and optical parameters at 785 nm of the four-layer model simulation.

	Layer	Layer thickness (mm)	$\mu_{a}$ (${\mathrm{mm}}^{-1}$)	$\mu_{s}^{\prime }$ (${\mathrm{mm}}^{-1}$)	$D_{b}$ (${\mathrm{mm}}^{2}/s$)
**Four-layer slab**	Scalp	3	0.019	0.726	$1\times 10^{-6}$
	Skull	7	0.014	0.946	$8\times 10^{-8}$
	CSF	2	0.001	0.002	$1\times 10^{-8}$
	Brain	∞	0.020	1.210	$6\times 10^{-6}$

We use $D_{b\_\text{brain}}$ and $D_{b\_\text{scalp}}$ to represent the Brownian diffusion coefficients in brain and scalp, respectively. We first simulated a pulsatile waveform of $D_{b\_\text{brain}}$ while gradually increasing $D_{b\_\text{scalp}}$ to show the model’s performance in recovering the $D_{b\_\text{brain}}$ waveform as well as its ability to identify the influence of $D_{b\_\text{scalp}}$. For the simulations, $D_{b\_\text{brain}}$ ranges from $3\times 10^{-6}\;{\mathrm{mm}}^{2}/s$ to $9\times 10^{-6}\;{\mathrm{mm}}^{2}/s$ follows a pulsatile pattern, whereas the $D_{b\_\text{scalp}}$ gradually increases from $5\times 10^{-7}\;{\mathrm{mm}}^{2}/s$ to $1.5\times 10^{-6}\;{\mathrm{mm}}^{2}/s$. Other parameters remain the same as in the baseline condition, and one level of noise (middle) was added to the simulated data, as described previously. For ${\mathrm{CBFi}}_{\text{baseline}}$ recovery, we simulated 1000 noise-added data samples under the baseline condition (Table 3). The mean values of the recovered CBFi from the DL model and the two-layer analytical fitting and the decorrelation speed measured using single-exponential fitting across the 1000 data samples were used as the baseline for calculating relative cerebral perfusion changes, respectively. rCBFi was calculated as $\mathrm{rCBFi}=\mathrm{CBFi}/{\mathrm{CBFi}}_{\text{baseline}}$.^7^^,^^27^

To quantify CBFi sensitivity, $D_{b\_\text{brain}}$ was varied by ±25% and ±50% relative to the baseline while maintaining $D_{b}$ in other layers constant. Similarly, $D_{b\_\text{scalp}}$ was varied by ±25% and ±50% relative to the baseline while keeping $D_{b}$ in other layers constant, allowing us to evaluate the model’s CBFi measurement sensitivity to scalp BFi (SBFi) changes. At each perturbation level, 1000 noise-added datasets were generated. The sensitivity is defined as^49^

$$S=\frac{\frac{\left(\mathrm{CBFi}-{\mathrm{CBFi}}_{0}\right)}{{\mathrm{CBFi}}_{0}}}{\frac{\left(D_{b}-D_{b0}\right)}{D_{b0}}}\times 100\%,$$ (11)

where CBFi and ${\mathrm{CBFi}}_{0}$ represent the recovered CBFi under perturbed and baseline conditions, respectively, $D_{b}$ and $D_{b0}$ denote the simulated brain or scalp Brownian coefficient at perturbation and baseline conditions. For single-exponential fitting, the numerator of Eq. (11) is replaced by the change in decorrelation speed. Ideally, a measured CBFi sensitivity close to 100% is preferred as it indicates accurate detection of cerebral blood flow changes. Conversely, a measured CBFi sensitivity to SBFi changes close to 0% suggests that the model is robust against SBFi variations, minimizing confounding effects from extracerebral blood flow.

We also calculated the recovered rCBF error by the DL model, two-layer analytical fitting, and single-exponential fitting, calculated using the below equation

$$\epsilon =(\mathrm{rCBF}-rD_{b})/rD_{b}\times 100\%,$$ (12)

where ϵ represents the percentage error in recovered relative flow change.

**Fig. 5:**
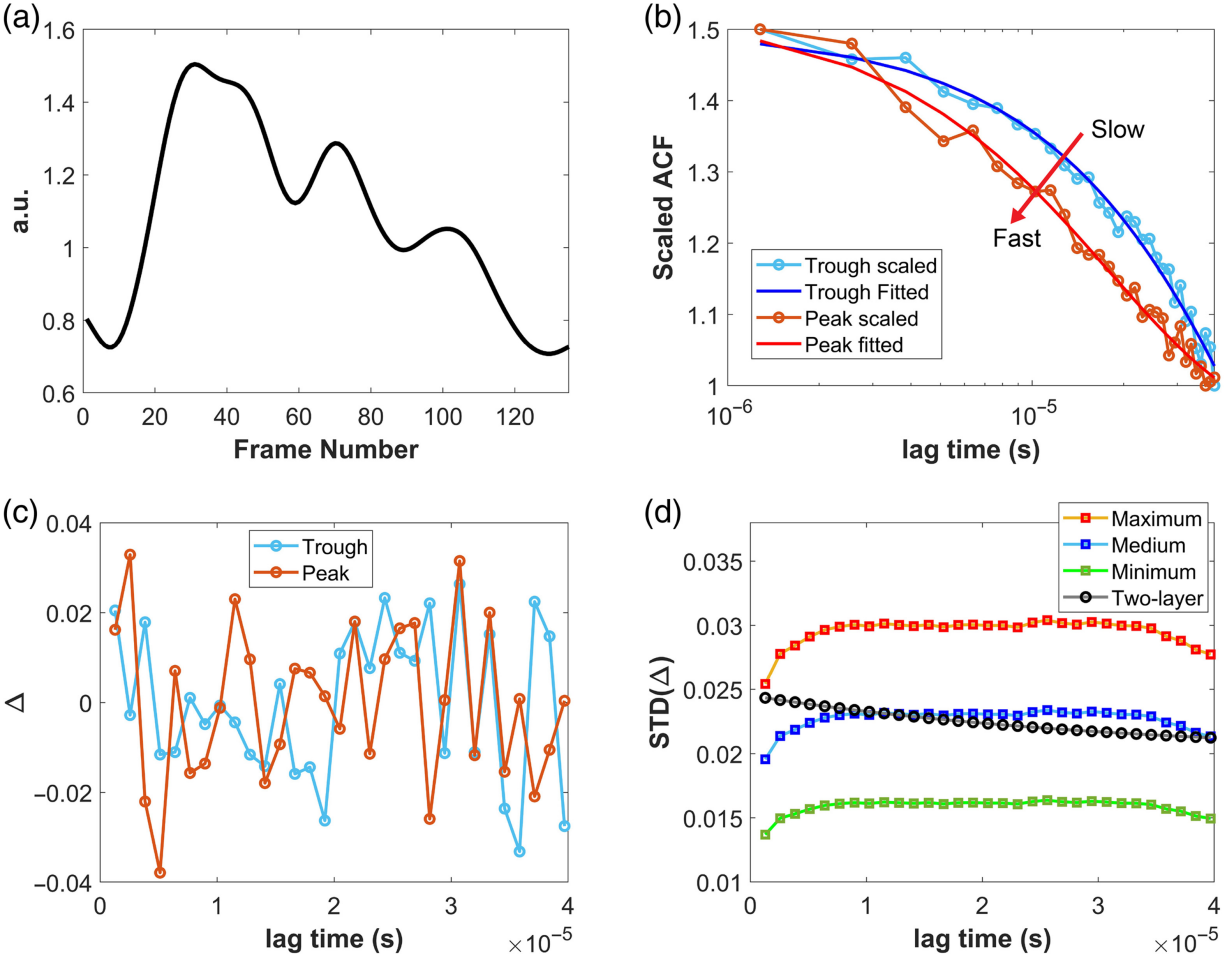
Examples of noise calculation. (a) A waveform of detected blood flow changes, where red and light blue circles indicate the peak and trough of one cycle. (b) Scaled SPAD ACF and the corresponding fitted single-exponential decay curves at the peak and trough. (c) The difference between the scaled SPAD ACF and the fitted single-exponential decay function at the peak and trough. (d) The standard deviation was calculated over 1000 frames across five tests; we scaled the original σ to get three levels of noise added to the clean dataset. The gray line with black circles represents the standard deviation used to generate the noisy dataset for the two-layer analytical model fitting; details can be found in the Supplementary Material.

For comparison, we added noise to the test dataset used for two-layer analytical model fitting. The clean and noise-added $g_{2}$ curves are shown in Fig. S1 of the Supplementary Material. The noise was generated using a widely accepted model from a previous study,^44^ employing a linear-τ sampling strategy.^45^ Details of the noise model and the specific parameters used are provided in the Supplementary Material. Specifically, we assumed a photon count rate of 3.3 kHz at a source-detector separation of ρ=35  mm and an integration time of 180 s. This photon count rate was estimated based on values reported in a prior study^39^ at ρ=30  mm and derived using the relationship of the unnormalized field autocorrelation function at zero time lag, $G_{1}(0)$.^50^^,^^51^ The noise level is shown in Fig. 5(d). For each perturbation level, we generated 1000 noisy ACFs for the two-layer analytical fitting. The dynamic parameters $D_{b}$ in the brain and extracerebral layers were treated as fitting variables, whereas the coherence factor β was assumed to be known and consistent with the value used in the four-layer model simulations (see the Supplementary Material).

### 2.6 Model’s Stability Test

To assess the model’s stability, we generated a series of datasets by varying the brain layer’s $\mu_{a}$, $\mu_{s}^{\prime }$, and the scalp and skull thicknesses using MC simulations based on the four-layer head model. Specifically, each parameter was assigned five evenly spaced values: $[0.005,0.010,0.015,0.020,0.025]\;{\mathrm{mm}}^{-1}$ for brain $\mu_{a}$; $[0.9,1.0,1.1,1.2,1.3]\;{\mathrm{mm}}^{-1}$ for brain $\mu_{s}^{\prime }$; [2, 3, 4, 5, 6] mm for scalp thickness ($L_{\text{scalp}}$); and [5, 6, 7, 8, 9] mm for skull thickness ($L_{\text{skull}}$). To compare the model’s stability in CBFi recovery, the dynamic parameters $D_{b}$ across all groups are same, as listed in Table 3. For rCBFi recovery, we simulated ±50% variations in $D_{b\_\text{brain}}$ with a baseline value of $D_{b\_\text{brain}}=6\times 10^{-6}\;{\mathrm{mm}}^{2}/s$. The group with $\mu_{a}=0.015\;{\mathrm{mm}}^{-1}$, $\mu_{s}^{\prime }=1.1\;{\mathrm{mm}}^{-1}$, $L_{\text{scalp}}=4\;\mathrm{mm}$, and $L_{\text{skull}}=7\;\mathrm{mm}$ was set as the reference group for calculating CBFi and rCBFi errors, defined as

$$E=(p-p_{\mathrm{ref}})/p_{\mathrm{ref}}\times 100\%,$$ (13)

where p denotes the recovered CBFi or rCBFi for a given parameter group and $p_{\mathrm{ref}}$ represents the recovered CBFi or rCBFi in the reference group. Not that when evaluating a parameter’s sensitivity, all other parameters were fixed at their reference values. The rCBFi recovery errors were calculated under two variations (−50% with $D_{b\_\text{brain}}=3\times 10^{-6}\;{\mathrm{mm}}^{2}/s$ and +50% with $D_{b\_\text{brain}}=9\times 10^{-6}\;{\mathrm{mm}}^{2}/s$). The final reported rCBFi error for each group is defined as the mean of the two results, helping to generalize the findings and avoid bias associated with the direction of variation. We used the errors in CBFi and rCBFi to characterize the model’s stability. An ideal model would yield errors close to 0, indicating consistent and reliable performance across parameter variations.

### 2.7 Human Physiological Response Tests

We first applied the proposed DL model, integrated with the SPAD sensor, to evaluate cerebral perfusion changes during a simple physiological response paradigm, expiratory breath-holding, which is expected to induce an increase in CBFi. The experiment was conducted on a healthy 29-year-old male subject. Each test session consisted of three phases: 30 s of normal breathing, followed by 30 s of expiratory breath-holding, and concluding with 30 s of resumed normal breathing. To assess the stability of our method, the test was repeated on three separate days.

Moreover, we conducted an additional test involving a routine activity, eating lunch, to evaluate digestion-induced changes in cerebral perfusion. CBFi measurements were recorded 30 min before the meal and at 5, 30, 75, and 120 min post-meal. In each test phase, 5000 frames of autocorrelation data were collected. As illustrated in Fig. 1, a custom 3D-printed probe was used to hold the source and detector fibers, which were attached to the participant’s forehead with an adjustable Velcro strap. During the tests, the power density from the source fiber tip was attenuated to remain below the maximum permissible exposure limit set by the American laser safety standard (28.5  mW for 785 nm laser).^52^ The participant wore laser safety goggles to prevent laser exposure to the eyes. Ethical approval was granted by the Biomedical Engineering Departmental Ethics Committee at the University of Strathclyde.

## 3 Results

### 3.1 Noise Characterization

Figure 5(a) shows an example of detected blood flow changes during a pulse cycle, recovered using single-exponential fitting. The fitting method is employed to illustrate how CBFi changes over a pulse cycle affect the measured signal. Figure 5(b) presents the scaled measured ACF and the corresponding fitted decay curves at the peak and trough in Fig. 5(a). Figure 5(c) displays the residuals, Δ=x(τ)−f(τ), calculated at the peak and trough at each of the 31 τ. Figure 5(d) is the standard deviation σ of the residuals averaged and scaled across the five test phases (each phase has 5000 frames of ACFs), which serves as the noise model for generating synthetic noise (see Sec. 2.2.).

We scaled the SPAD ACF data before noise characterization [using Eq. (9)] to match the training input format. Because the SPAD’s autocorrelator uses linear lag spacing, the noise standard deviation is expected to follow an exponential decay.^45^ Indeed, in Fig. 5(d), the standard deviation is lower at the first and last lag points compared with the middle; we believe this occurs due to our scaling and the rapid decay of the $g_{2}$ curve over the initial lag range at high flow rates. In addition, as the correlation time ranges from 1.28 to 39.68  μs, corresponding to the very beginning of a full $g_{2}$ decay, so the correlation values drop off very rapidly (especially for high flow). Consequently, the first point of the scaled SPAD ACF is 1.5 (by design of the scaling).

### 3.2 CBFi Waveform Recovery on Simulated Data

**Fig. 6:**
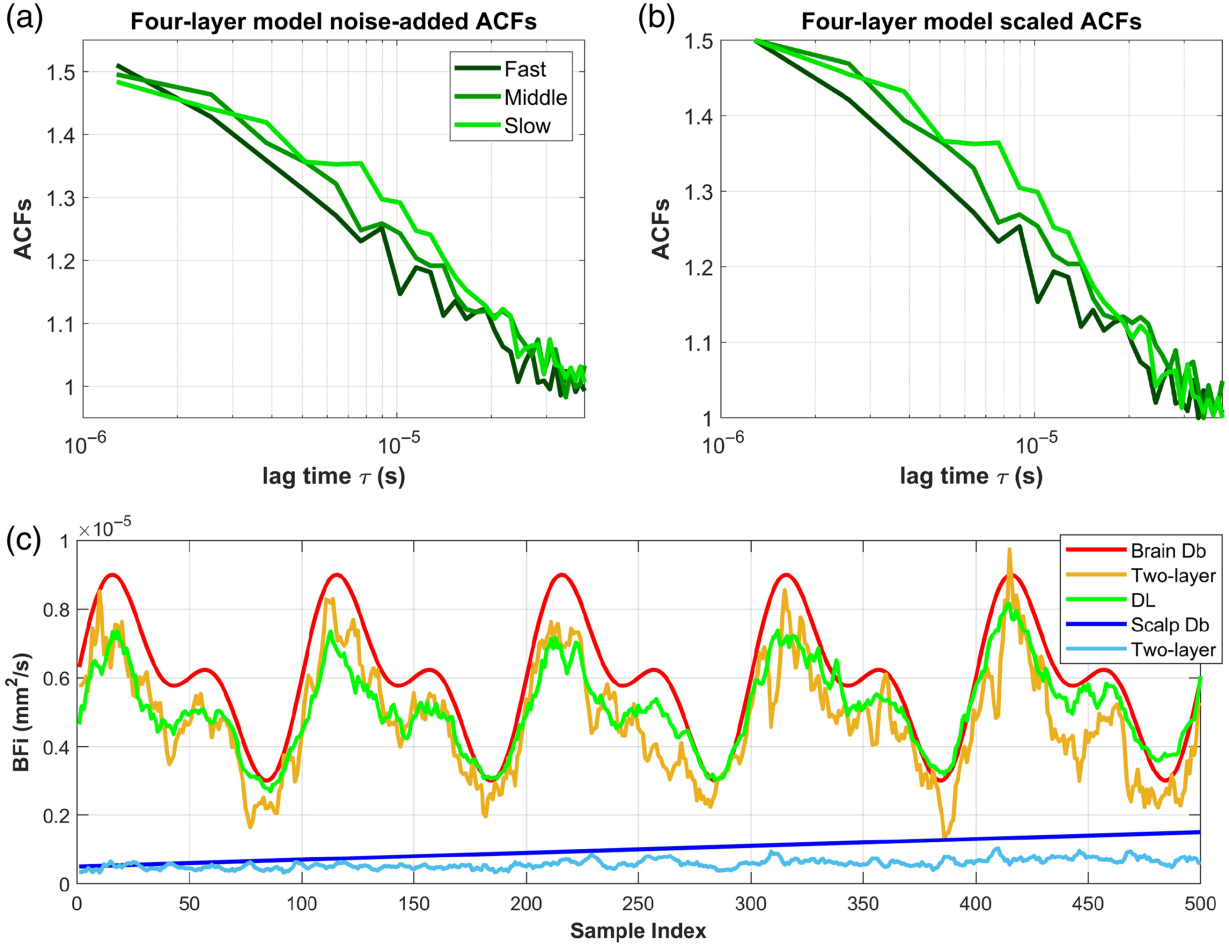
(a) Representative noise-added ACFs from the test dataset simulated with the four-layer head model. (b) Scaled DL format ACFs ready for input to the model for CBFi estimation. Note that for the two-layer analytical fitting, the used noise model has been introduced in Sec. 2.5. (c) Pulsatile CBFi waveform recovered from the test dataset compared with the two-layer analytical model fitting. All displayed curves were smoothed in MATLAB (function: *smooth()*, default method: moving average, span = 5). The green curve represents the CBFi waveform estimated by the DL model, whereas the yellow curve represents CBFi recovered by the two-layer analytical fitting. The red curve denotes the simulated brain $D_{b}$ (ground truth), the blue line corresponds to the simulated scalp $D_{b}$, and the light blue curve is the recovered extracerebral BFi by the two-layer analytical fitting.

In this section, we first visualized the performance of the proposed DL model in CBFi waveform recovery and assessed its ability to isolate SBFi variations, in comparison with the two-layer analytical fitting. Figures 6(a) and 6(b) show the representative ACFs from the four-layer model simulation and are used as input for the trained DL model. Figure 6(c) presents the recovered CBFi waveform from the simulated dataset of the four-layer slab head model. The results show that the DL can effectively reconstruct CBFi waveforms and achieve accuracy comparable to the two-layer analytical fitting, although both methods tend to underestimate absolute CBFi values. This underestimation arises from the two-layer analytical model’s bias, where the scalp and skull are grouped into a single extracerebral layer. As blood flow in the skull is typically minimal, this grouping leads to a downward bias in CBFi estimation as well as SBFi estimations.^22^^,^^38^ The results presented in Fig. 6 align with the two-layer analytical model-induced bias, suggesting that our model has learned similar characteristics of the analytical model in terms of absolute CBFi recovery. As the simulated SBFi was programmed to increase linearly (from $5\times 10^{-7}$ to $1.5\times 10^{-6}\;{\mathrm{mm}}^{2}/s$), the DL recovered CBFi waveform also exhibits a slight upward shift with increasing sample index (time). This suggests that the recovered CBFi remains partially influenced by blood flow changes in the shallow layers (i.e., not perfectly separating scalp influence), whereas the two-layer analytical fitting tends to show a more stable ability to isolate the scalp flow influence. The quantitative analysis of CBFi sensitivity to both CBFi and SBFi changes is provided in Secs. 3.3 and 3.4.

**Fig. 7:**
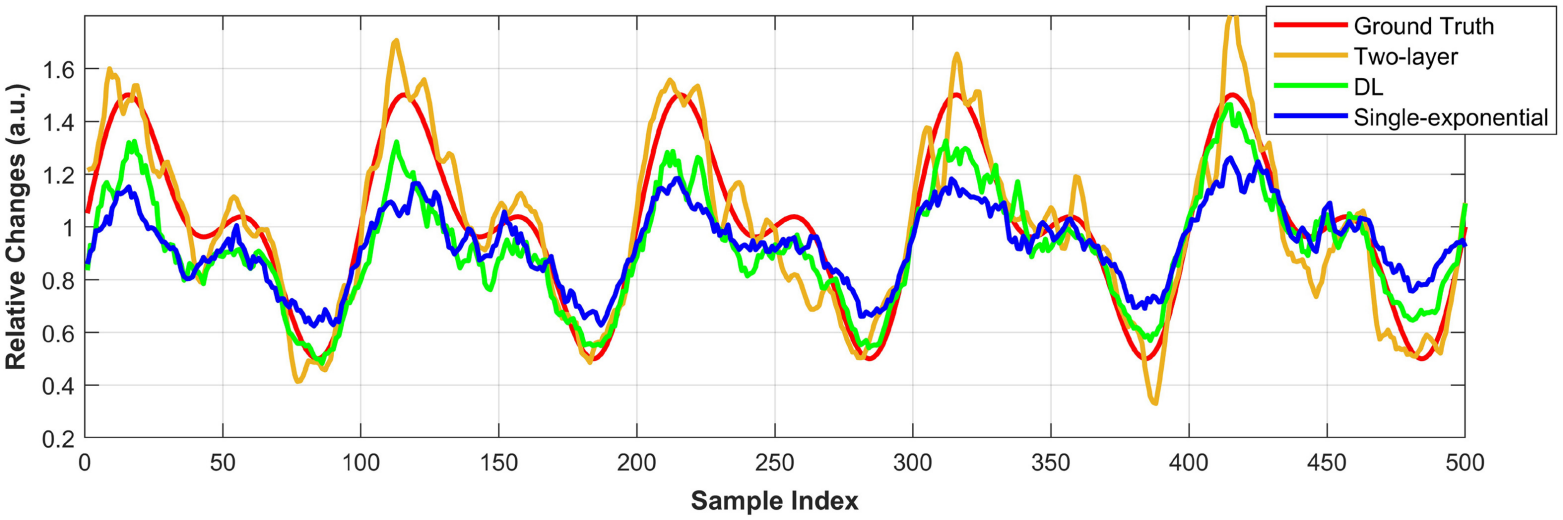
Recovered relative CBF changes using the two-layer analytical fitting (yellow line), DL model (green line), and single-exponential fitting (blue line) on the simulated dataset, with a smoothing function applied. The red line denotes the simulated ground truth.

Figure 7 presents the recovered rCBFi using the proposed DL model, the two-layer analytical fitting, and the relative changes in decorrelation speed obtained via single-exponential fitting. Please note that the single-exponential fitting was applied to the scaled ACFs formatted for the DL model. We have verified that this scaling has a negligible impact on the recovery of relative decorrelation speed (validation results are provided in Fig. S4 of the Supplementary Material).

Figure 7 shows that the two-layer analytical fitting most accurately recovers rCBFi relative to the ground truth. Although both the DL model and the single-exponential fitting capture the trend of relative rCBFi changes, they tend to underestimate the magnitude of these changes. Among the two, the DL model provides a closer approximation to the ground truth than the single-exponential fitting. In addition, compared with the two-layer analytical fitting, both the DL model and single-exponential fitting show an increasing trend in recovered relative changes as SBFi increases, suggesting that they are influenced by SBFi variations. It is worth noting that fitting the 500 data samples using the two-layer analytical model required ∼6.7  h (see the Supplementary Material for fitting details). The quantitative analysis and comparison of these recovered relative blood flow changes are provided in Secs. 3.3 and 3.4.

### 3.3 Model’s Brain Sensitivity

**Fig. 8:**
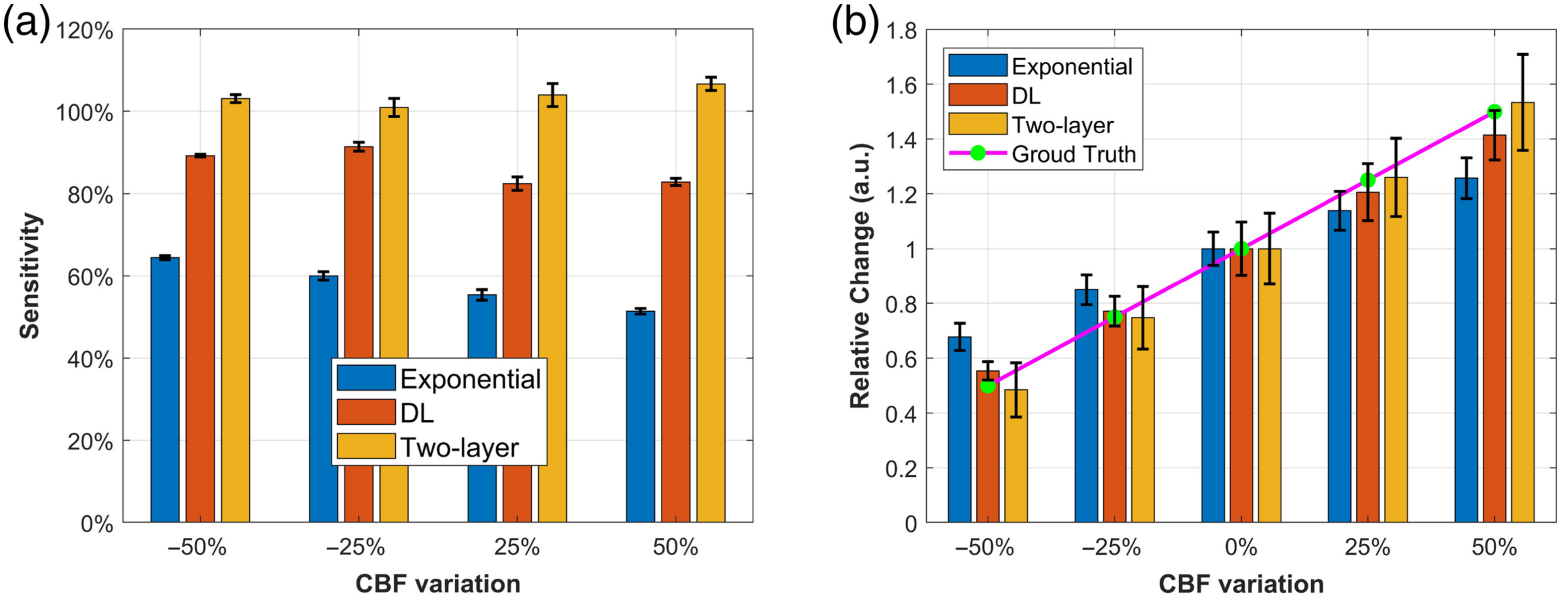
(a) and (b) Bar charts showing the recovered brain sensitivity and relative changes in response to CBFi variations are presented for single-exponential fitting, the DL model, and the two-layer analytical fitting. Note: We applied nonparametric bootstrapping to quantify the uncertainty in the estimated mean values of the sensitivity data as the original standard deviations were relatively large, making it difficult to compare results across different methods. Specifically, we randomly resampled 1000 data points from the original sensitivity data and calculated their mean. This process was repeated 1000 times, resulting in a distribution of bootstrap means. The error bars in panel (b) represent the 25th and 75th percentiles of the recovered data, providing an interquartile range-based measure of variability.

In this section, we quantified the model’s brain sensitivity calculated using Eq. (11). Baseline CBFi was obtained using the mean value of the recovered CBFi from 1000 noise-added data. As described in Sec. 2.4, CBFi was varied by ±25% and ±50% relative to the baseline. The results were visualized using bar graphs, as shown in Fig. 8. The bar heights represent the mean of the recovered data, and the error bars indicate the standard error of the mean, estimated using nonparametric bootstrapping (1000 resamples). This approach facilitates a more statistically robust comparison of central trends across conditions by quantifying uncertainty in the mean. Figure 8(a) shows that the proposed DL model demonstrates higher sensitivity to CBFi variations across all levels compared with single-exponential fitting, although neither method reaches full sensitivity (100%). On average, the DL model achieves a CBFi sensitivity of 86.5%, whereas single-exponential fitting reaches only 57.7%. By contrast, the two-layer analytical fitting exhibits superior performance, achieving an average sensitivity of 103.6%. Overall, the DL model improves CBFi sensitivity by ∼50% compared with single-exponential fitting, although it still falls short of the accuracy provided by the two-layer analytical approach.

Figure 8(b) presents the recovered CBF changes across different variation levels. The DL model demonstrates more accurate recovery of relative changes compared with single-exponential fitting, with an average relative error of 4.1%, versus 12.7% for the latter, as calculated using Eq. (12). Both methods tend to underestimate the true CBF changes. By contrast, the two-layer analytical fitting yields the most accurate results, with the smallest average error of 1.2%, consistent with the findings shown in Fig. 7. In addition, in Fig. 8(b), the error bars at different $D_{b\_\text{brain}}$ variation levels indicate that the DL model has a lower standard deviation than fitting when $D_{b\_\text{brain}}$ is smaller than the baseline. However, the opposite trend is observed when $D_{b\_\text{brain}}$ is larger than the baseline. This suggests that the proposed DL model produces more consistent estimates (i.e., lower variance) at low flow rates but exhibits greater variability at higher flow rates compared with single-exponential fitting. This is likely due to the rapid decay of the autocorrelation function at high flow rates, which causes a loss of informative features in the measured ACF due to the limited time lag range. As a result, the model tends to underestimate flow under these conditions.

### 3.4 Model’s Ability to Separate Extracerebral Blood Flow Confounder

**Fig. 9:**
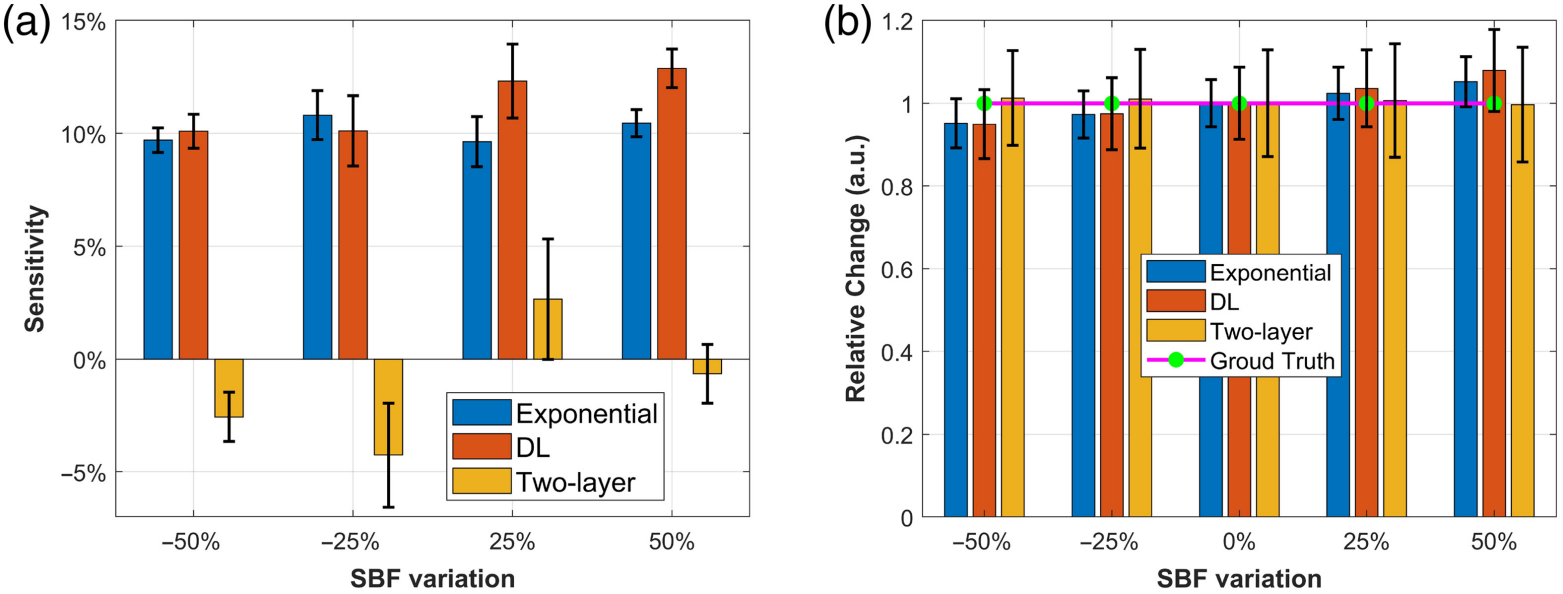
(a) and (b) Bar charts showing the recovered brain sensitivity and relative change in response to SBFi variation for single-exponential fitting, DL model, and the two-layer fitting. The error bars in panel (a) were calculated using the bootstrap distribution of mean values, following the same procedure used for the CBF sensitivity analysis. The error bars represent the 25th and 75th percentiles of the recovered data.

Figure 9(a) shows the recovered CBFi sensitivity to SBFi. The proposed DL model exhibits scalp sensitivity comparable to that of single-exponential fitting, with average sensitivities of 11.4% and 10.2%, respectively. By contrast, the two-layer analytical fitting shows an average sensitivity of −1.3%, where the negative value [as defined by Eq. (11)] indicates an inverse relationship between CBFi and changes in SBFi.

For rCBFi recovery, the results are presented in Fig. 9(b). All three methods demonstrate relatively good accuracy, with average errors of 3.8% for the DL model, 3.1% for single-exponential fitting, and 0.7% for the two-layer analytical fitting, based on Eq. (12). These results suggest that both the DL model and single-exponential fitting are similarly effective at minimizing the influence of extracerebral layer changes when estimating CBF, whereas the two-layer analytical model achieves the highest accuracy but may produce a slight inverse response.

It is also worth noting that the error bars in both figures indicate the DL model exhibits a slightly larger standard deviation compared with single-exponential fitting, implying that the DL estimates are somewhat more variable in response to changes in scalp blood flow.

### 3.5 Model’s Stability Test

**Fig. 10:**
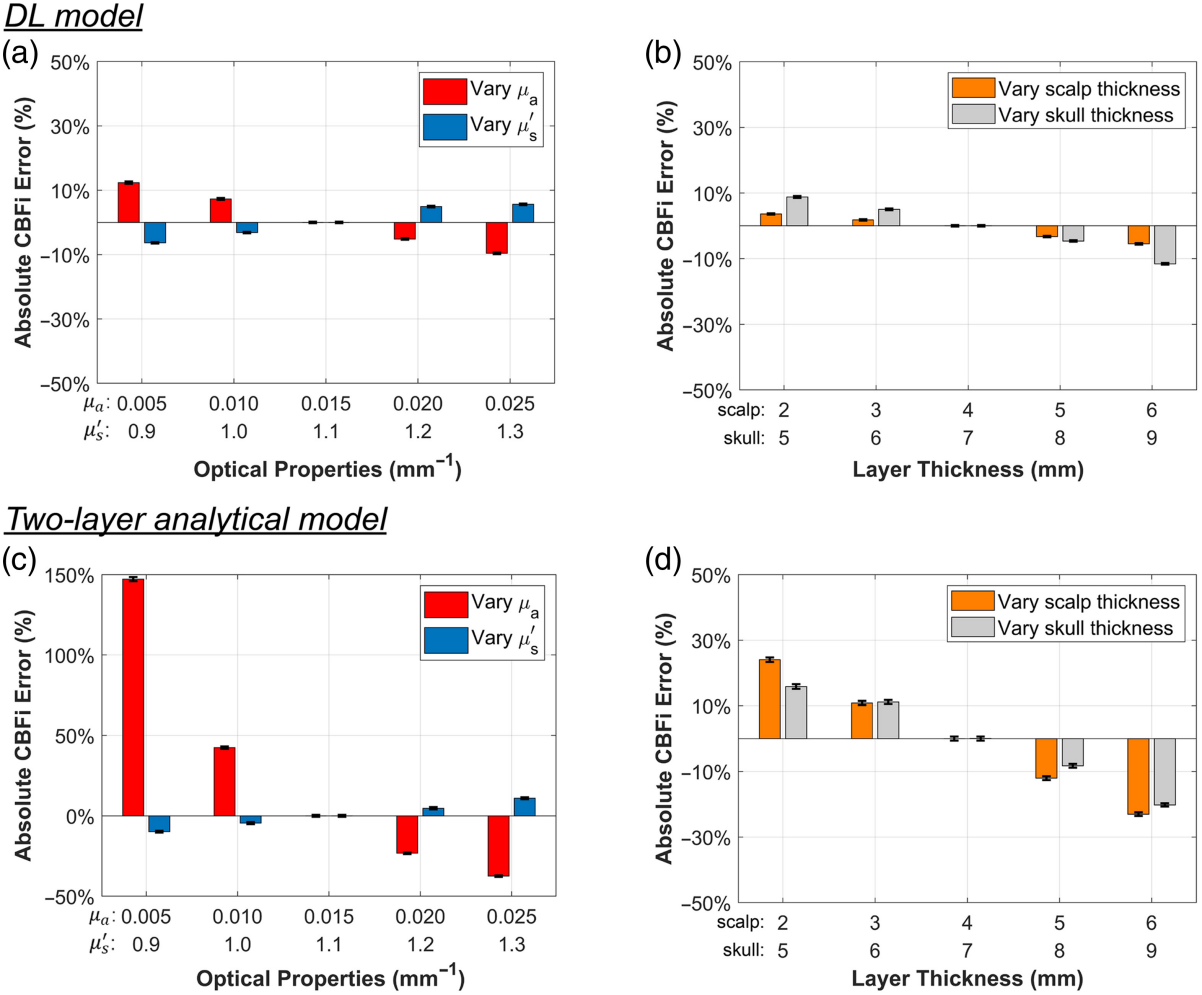
Comparison of absolute CBFi recovery between the DL method and the two-layer analytical model fitting. Bootstrapping was applied to estimate the mean CBFi errors from 1000 data points, following the same procedure described in the previous section. CBFi errors were calculated using Eq. (13). When varying one parameter, all other parameters were fixed at their baseline values ($\mu_{a}=0.015$, $\mu_{s}^{\prime }=1.1$, $L_{\text{scalp}}=4\;\mathrm{mm}$, and $L_{\text{skull}}=7\;\mathrm{mm}$). These baseline values were also assumed in the two-layer analytical model fitting for consistency.

In this section, we compared the stability of the proposed DL model with that of the two-layer analytical model. The dataset used for the two-layer analytical fitting was the same, as described in Sec. 2.6, and noise was added as outlined in the Supplementary Material. Absolute CBFi and rCBFi errors were calculated using Eq. (13). Figure 10 presents the absolute CBFi recovery errors for the DL method and the two-layer analytical fitting. The DL model consistently demonstrates lower CBFi errors across variations in brain layer $\mu_{a}$, $\mu_{s}^{\prime }$, $L_{\text{scalp}}$, and $L_{\text{skull}}$, suggesting improved robustness. These results demonstrate that the DL model maintains good stability across different parameter settings.

**Fig. 11:**
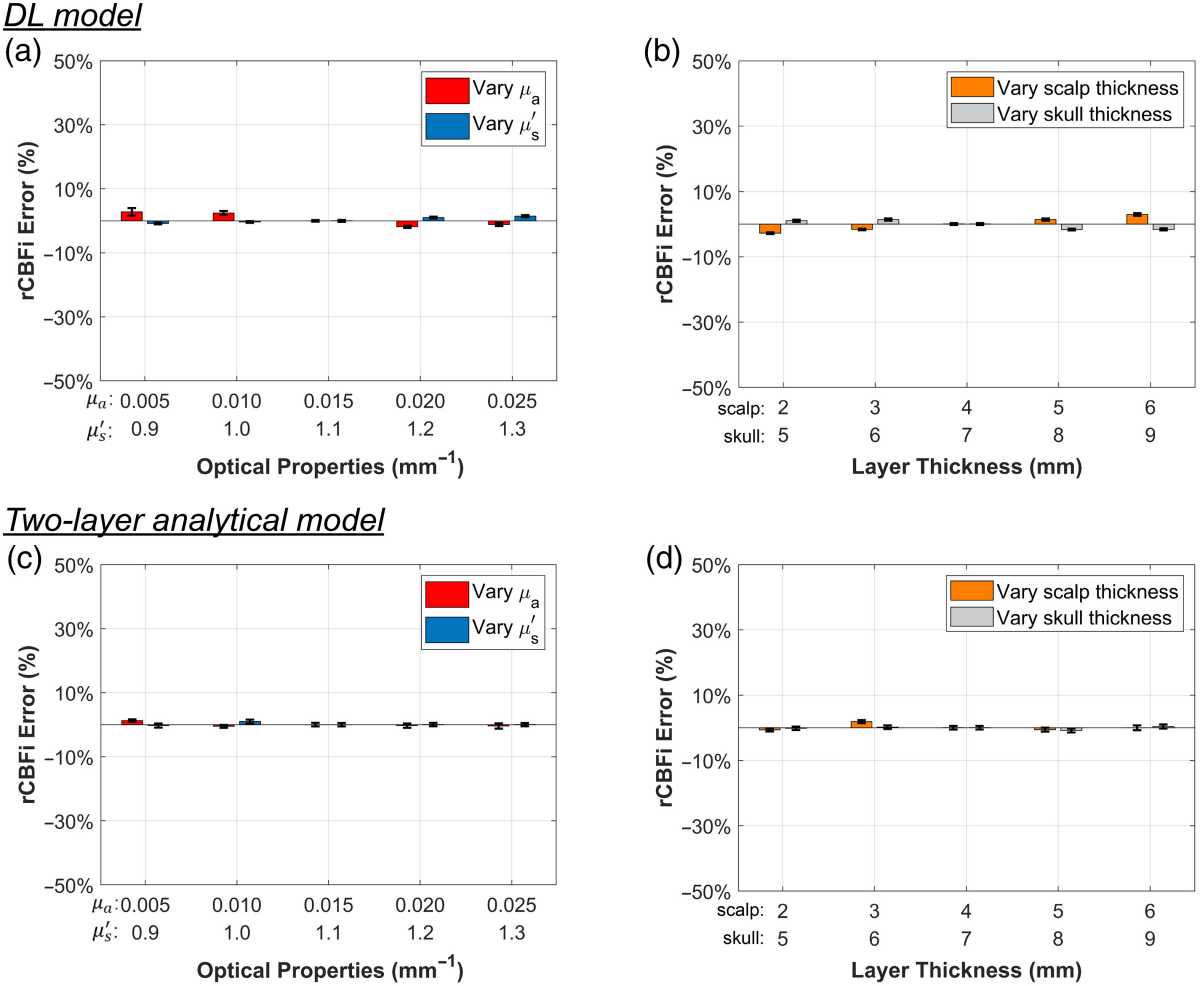
Comparison of rCBFi recovery between the DL method and the two-layer analytical model fitting. The same strategy used in Fig. 10 for absolute CBFi comparison was applied here. rCBFi was calculated based on ±50% perturbations relative to the baseline condition. The displayed rCBFi errors were computed using Eq. (13), with the errors from the two perturbation levels averaged to remove bias related to the direction of variation.

In Fig. 11, the DL model shows comparable stability in rCBFi recovery relative to the two-layer analytical model, although the latter achieves slightly lower rCBFi errors. These results indicate that both methods are capable of accurately recovering rCBFi across varying tissue parameters.

### 3.6 Baseline Blood Flow Waveform Recovery

**Fig. 12:**
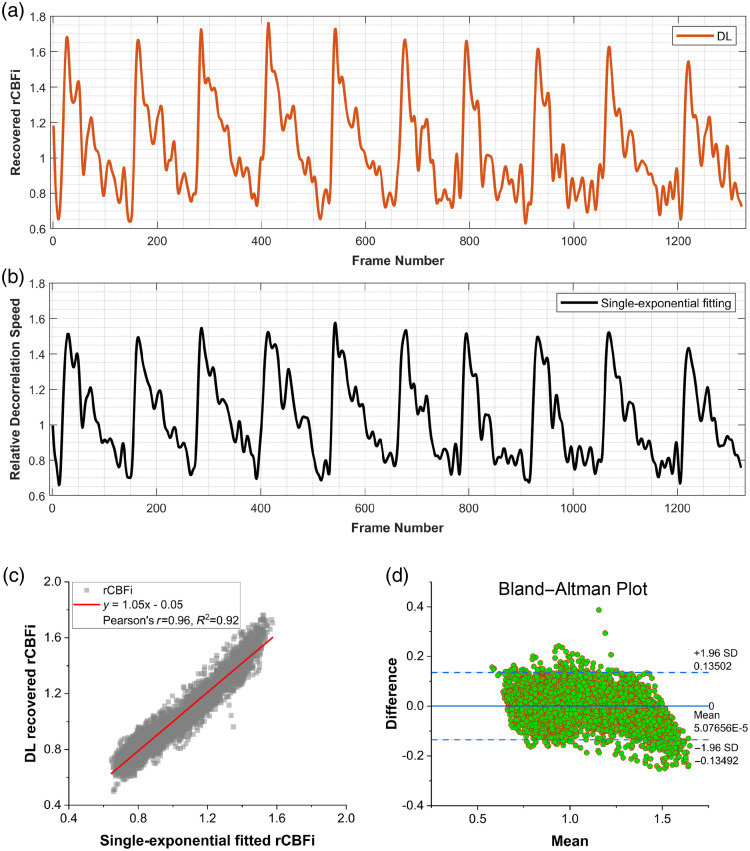
Recovered baseline cerebral perfusion waveform (30 min before lunch). (a) and (b) The rCBFi waveform was filtered using a third-order Butterworth low-pass filter (cutoff frequency = 0.15, normalized) with zero-phase filtering (filtfilt) to reduce high-frequency noise. The mean value of recovered CBFi by DL and the mean value of recovered decorrelation speed by single-exponential fitting were taken as the baselines to calculate the relative cerebral perfusion changes, respectively. (c) Pearson’s correlation analysis and (d) Bland–Altman analysis between the DL model and the single-exponential fitting recovered the CBF waveform.

In this section, we evaluated the recovered cerebral perfusion waveform using the proposed DL model under the resting-state (5000 frames) in the subject. Figure 12 presents the DL-recovered and single-exponential fitting-recovered cerebral perfusion waveforms at the baseline; we applied a third-order Butterworth low-pass filter (cutoff frequency = 0.15, normalized) with zero-phase filtering for the waveform recovery. Pearson’s correlation analysis showed a strong linear relationship between the two methods (r=0.96, $R^{2}=0.92$, n=5000), indicating that our model closely matches the waveform recovered by the traditional curve-fitting method. The Bland–Altman plot in Fig. 12(d) further illustrates the consistency between the two methods. The mean difference is close to 0 ($5.08\times 10^{-5}$), and 95% of the differences lie within the limits of agreement (±1.96 SD), ranging from −0.13492 to 0.13502. These results confirm a high level of agreement and minimal systematic bias between the DL and single-exponential methods for rCBFi estimation.

### 3.7 Repeated Breath-Holding Tests and a Lunch Test on a Healthy Male Adult

**Fig. 13:**
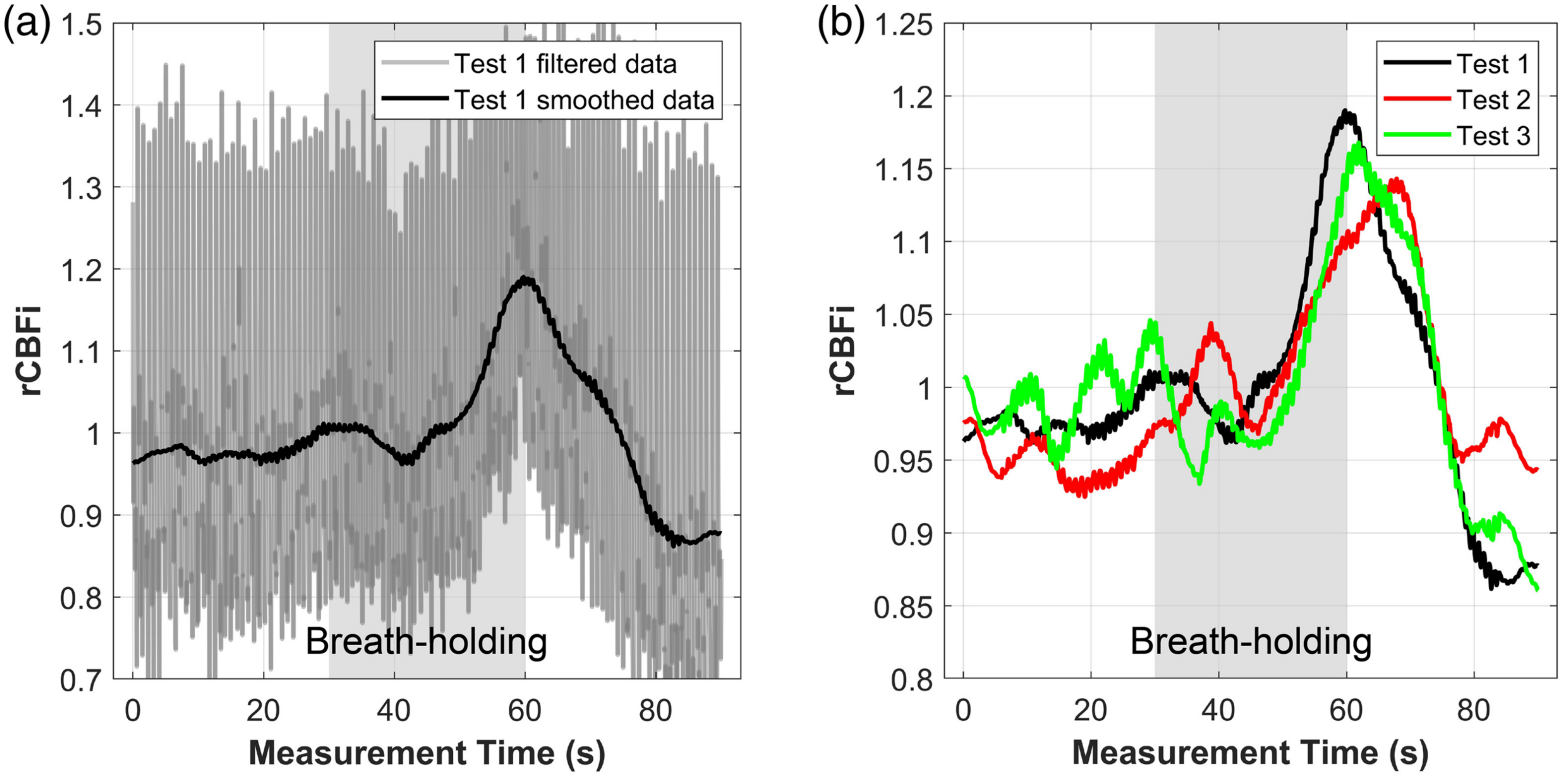
Breath-holding test results on a healthy male adult, repeated 3 times. (a) A representative recovered rCBFi (test 1) using our DL model; the semi-transparent gray curve is the filtered (see Fig. 12) blood flow waveform during the whole test session; the solid black curve is the smoothed rCBFi using a Savitzky–Golay filter (window = 1001, order = 3). (b) The subject’s rCBFi response to the breath-holding test, repeated 3 times (tested on different days, without practice before testing). The first 30 s is normal breath, the second 30 s is an expiratory breath hold, and the last 30 s is recovering back to normal breath.

Figure 13 presents the results of the subject’s response to breath-holding, recovered using the proposed DL model. The test was repeated 3 times on different days without prior practice. In Fig. 13(a), a representative example from test 1 is shown, where the semi-transparent gray curve represents the filtered rCBFi signal (same as used in Fig. 12), and the solid black line indicates the smoothed waveform obtained using a Savitzky–Golay filter (window = 1001, order = 3). The smoothed curve clearly captures a rise in rCBFi during the breath-holding phase (30 to 60 s), followed by a return to baseline during the recovery phase (60 to 90 s), consistent with the expected physiological response. Figure 13(b) displays the rCBFi waveforms from all three repeated trials, showing cerebral blood flow increases of ∼21.2%, 20.3%, and 17.8% at the end of the breath-holding phase for tests 1, 2, and 3, respectively. Despite day-to-day variability, all three tests consistently show an increase in rCBFi during the breath-holding period, reflecting elevated cerebral perfusion in response to hypercapnia. These results demonstrate the intra-subject repeatability of the DL model in capturing dynamic cerebral blood flow changes induced by breath-holding.

**Fig. 14:**
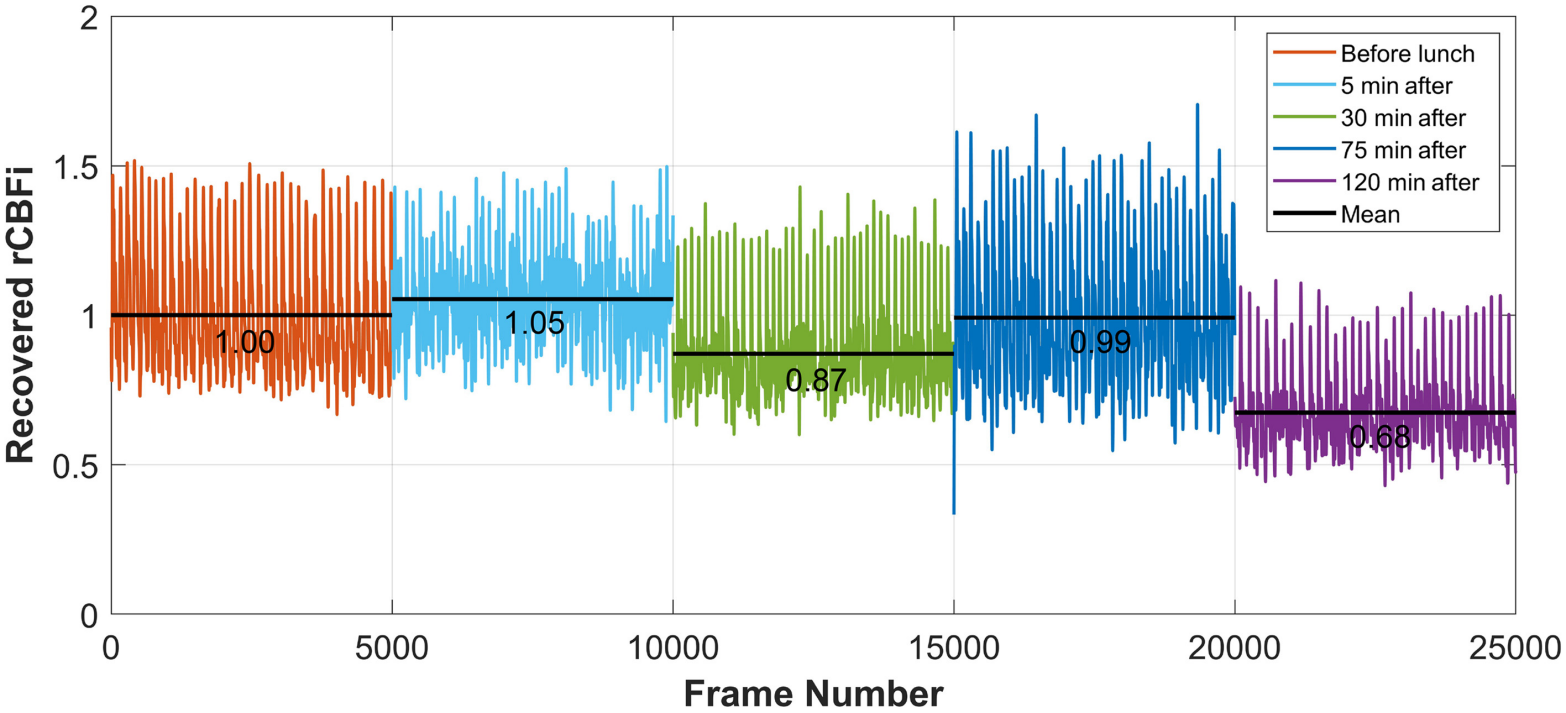
Cerebral perfusion monitoring using the proposed DL model during the lunch test. The black lines represent the mean values of the recovered rCBFi at each test phase. The mean value from the first test phase (30 min before lunch) was used as the global baseline for rCBFi calculation.

Figure 14 presents cerebral perfusion changes recovered by our model during the lunch test. The test conducted 30 min before lunch was used as the global baseline for calculating rCBFi at subsequent test phases. The results indicate a slight increase in cerebral perfusion immediately after lunch (5 min post-meal, light blue curves in Fig. 14), likely due to cortical stimulation from gustatory stimulation.^53^^,^^54^ At 30 min, as blood flow is redirected to digestion, a short-term cerebral perfusion decrease occurred, followed by a return to baseline at 75 min (end of postprandial hyperemia^55^). Interestingly, at 120 min after lunch, a notable decrease was observed, coinciding with the subject’s reported fatigue and drowsiness.^56^

In addition, we evaluated the computational efficiency of the DL model. The average processing time per 5000 frames was 0.06 s, using our workstation GPU, compared with 44.98 s for single-exponential fitting on our workstation CPU (CPU: Intel(R) Core™ i9-10900X at a rate of 3.70 GHz; Memory: 128 GB; GPU: NVIDIA Quadro RTX 5000). This represents a 750-fold speed improvement, making our approach more suitable for neurophotonics applications where continuous monitoring and fast feedback are needed (e.g., bedside CBF tracking or neurofeedback).

## 4 Discussion

We successfully implemented a DL model trained on the two-layer DCS analytical model-generated dataset with noise calculated from subject-specific baseline measurements. Our model demonstrated improved CBFi sensitivity of 86.5% versus 57.7%, rCBFi error of 4.1% versus 12.7% on the simulated dataset, and 750× faster than the single-exponential fitting method during the physiological response test. Compared with the two-layer analytical fitting, our model exhibited greater stability in absolute CBFi recovery. Notably, the two-layer analytical model showed a CBFi sensitivity exceeding 100%, indicating an overestimation of CBF changes, i.e., the recovered rCBFi fluctuated more than the ground truth (Figs. 7 and 8). In our analysis, the scalp and skull were grouped as the extracerebral layer, whereas the CSF and brain were grouped as the cerebral layer. This anatomical simplification may have introduced model mismatch when applying the two-layer analytical model to more complex head geometries. The impact of this grouping strategy on sensitivity and recovery accuracy will be further examined in future work.

Although our model showed excellent performance, it is worth noting that single-exponential fitting also performed well in our study, significantly enhancing CBF sensitivity and effectively minimizing the influence of the scalp layer relative to older conventional DCS setups (Fig. 9). This improvement can be attributed to advances in SPAD sensor technology, which enables early time lag detection while substantially improving the measurement SNR.^18^^–^^20^^,^^57^ Single-exponential fitting, a simplified implementation of the semi-infinite analytical solution, is commonly used to characterize relative CBF changes.^13^ As the early part of the $g_{2}$ curve is primarily influenced by brain blood flow, whereas the later part is dominated by scalp blood flow, fitting only the early portion of the $g_{2}$ curve can enhance brain blood flow sensitivity while reducing scalp interference.^6^^,^^58^

Indeed, a primary limitation of this work is also the restricted time lag range. The two-layer analytical model has demonstrated its ability to separate CBF and extracerebral blood flow (Figs. 8 and 9). However, during training dataset preparation, the time lag range was tailored to 1.28 to 39.68  μs to match the SPAD settings, which resulted in the loss of some dynamic information from shallow layers. By training only on the early portion of the $g_{2}$ curve, we provided the network with less information about the slow decays from shallow flow, making it harder for the model to learn to distinguish extracerebral contributions. This limitation also explains why the DL model accurately recovers CBFi at lower flow rates but underestimates CBFi (Fig. 6) and rCBFi (Fig. 7) at higher flow rates. As BFi increases, the $g_{2}$ curve shifts leftward and decays faster within the limited lag window; the restricted time lag range may not capture sufficient deep tissue information. In short, there is a trade-off between brain sensitivity and the model’s ability to isolate extracerebral confounders in our current system.

Moreover, as BFi decreases or ρ becomes smaller, the $g_{2}$ curve shifts to the right, resulting in a plateau across the entire measured lag time range. Consequently, the limited lag time range constrains the applicability of our system for small ρ measurements or for detecting slow blood flow (i.e., when the decorrelation time exceeds ∼40  μs). This constraint renders the current DL model suitable only for the specific lag time range used in the system.

Regarding noise characterization in SPAD experiments, our approach provides an approximate representation of real noise. Noise levels can vary based on the detection region, source power, and inter-subjects, which means that including a broader range of noise levels would improve robustness to experimental variations. However, due to computational constraints, only three noise levels were incorporated into our dataset. In addition, ρ was fixed at 35 mm for subject-specific calibration, and the optical properties ($\mu_{a}$ and $\mu_{s}^{\prime }$) in the extracerebral layer were fixed. Expanding the range of ρ and sampling more extensively across other model parameters would enable broader coverage of application scenarios using the proposed method, supporting both longitudinal studies and inter-subject comparisons. We also applied a min–max scaling to both experimental and simulated data. This ensured matching ranges but could alter the shape of the ACF curve slightly (caused by noise), effectively introducing a small systematic difference between how labels were generated and how experimental data appear. In future work, more sophisticated normalization or data augmentation strategies could be explored to bridge this gap. In the breath-holding test, our method effectively captured cerebral perfusion changes induced by expiratory breath-holding. The test was repeated 3 times on the same subject, demonstrating strong intra-subject repeatability and highlighting the method’s reliability for longitudinal monitoring. To further evaluate the system’s capability for inter-subject CBF monitoring, additional breath-holding tests were conducted on two other healthy male adults. The results are presented in Fig. S5 in the Supplementary Material.

Although the system successfully detected relative changes in cerebral perfusion during breath-holding in both subjects, the magnitude and waveform patterns of the responses varied significantly. Moreover, in contrast to the subject used to derive the noise model, the system failed to reconstruct a clear pulsatile baseline CBF waveform in these two subjects. As discussed previously, this limitation is likely due to the subject-specific nature of the noise model used during training, which may be too narrow to generalize effectively across individuals. Developing a more robust and generalizable noise model is therefore a key direction for improving the adaptability and performance of the current system.

During the lunch test, we observed slight fluctuations in recovered rCBFi curves across different test phases. As these tests were conducted separately at different time points, variations in noise due to hardware instability may have contributed to these fluctuations. Factors such as slight differences in probe positioning, applied pressure, and detected photon count across trials could affect measurements as well. Besides, the SPAD array is highly sensitive to movement, vibration, breathing, and airflow in the test environment, all of which could introduce measurement instability. Increasing the number of noise levels in future studies may help address this issue. Moving forward, we will implement additional noise levels and optimize probe design to reduce motion and pressure variations in real-world applications. This data-driven approach can be further enhanced by incorporating additional measurement modes (e.g., multidistance or time-resolved DCS) to provide the model with richer information for separating tissue layers.

The proposed SPAD-DCS integrated with the DL method holds the ability to noninvasively monitor cerebral blood flow with high temporal resolution, which has broad neurophotonics implications. Competing modalities (fMRI, PET) are too slow or impractical for continuous monitoring. Traditional DCS is promising but struggles with accuracy at deep layers. The contributions of this work (layer-aware DL model + high-SNR SPAD) address those limitations head-on, bringing DCS closer to a viable neuro-monitoring tool for brain health. For example, real-time bedside monitoring in neurocritical care, neurovascular coupling studies, or augmented neuroimaging combined with functional near-infrared spectroscopy.

## 5 Conclusion

In this work, we demonstrated the feasibility and advantages of using a DL model based on the two-layer DCS analytical model, combined with an SPAD sensor for CBFi monitoring. The proposed DL model significantly improves CBFi sensitivity and rCBFi accuracy while exhibiting a comparable ability to an early-lag single-exponential fitting in minimizing superficial layer influence. In addition, we applied this approach to evaluate physiological responses and demonstrated its utility in monitoring CBF changes in a healthy subject. With further hardware improvements (e.g., wider lag ranges, faster readouts), more extensive noise modeling, and expanded training datasets (including more noise levels), we anticipate even more accurate and robust performance in the future.

## Supplementary Material

10.1117/1.NPh.12.3.035008.s01

## Data Availability

Data underlying the results presented in this paper are not publicly available at this time but may be obtained from the authors upon reasonable request.
