# Supplementary material for: Cerebral blood flow monitoring using a deep learning implementation of the two-layer diffuse correlation spectroscopy analytical model with a 512 × 512 SPAD array
Source: Neurophotonics. 2025 Aug 18;12(3):035008. doi: 10.1117/1.NPh.12.3.035008 (PMC12360787; doi:10.1117/1.NPh.12.3.035008)
Supplement: Supplementary file 1 [file NPh_012_035008_SD001.pdf]

Supplementary Material for:
Cerebral blood flow monitoring using a deep learning implementation of the two-layer DCS analytical model with a 512×512 SPAD array

Mingliang Pan,[a] Chenxu Li,[a] Yuanzhe Zhang,[a] Alan Mollins,[a] Quan Wang,[a] Ahmet T. Erdogan,[b] Yuanyuan Hua,[b] Zhenya Zang,[a] Neil Finlayson,[b] Robert K. Henderson,[b] David Day-Uei Li[a,*]

[a]University of Strathclyde, Department of Biomedical Engineering, Glasgow, UK
[b]University of Edinburgh, School of Engineering, Integrated Nano and Micro Systems (IMNS), Edinburgh, UK

*David Day-Uei Li, E-mail: David.li@strath.ac.uk

1. Noise model and fitting method for the two-layer analytical model

To add noise to the four-layer model simulated test dataset for the two-layer analytical fitting, we employed the Gaussian noise model with zero mean and a standard deviation based on the noise model proposed by Zhou *et al.* to our simulations. The standard deviation is defined as:

$$\sigma(\tau) = \sqrt{\frac{T_{\text{bin}}}{T_{\text{int}}}}\left[\beta^2 \frac{(1+e^{-2\Gamma T_{\text{bin}}})(1+e^{-2\Gamma\tau}) + 2m(1-e^{-2\Gamma T_{\text{bin}}})e^{-2\Gamma\tau}}{1-e^{-2\Gamma T_{\text{bin}}}} + \frac{2\beta(1+e^{-2\Gamma T_{\text{bin}}})}{\langle n\rangle} + \frac{1+\beta e^{-\Gamma\tau}}{\langle n\rangle^2}\right]^{1/2}, \quad (1)$$

where Γ is the decay rate, extracted by fitting the simulated $g_2(\tau)$ curves with a single exponential decay model: $g_2(\tau) = 1 + \exp(-2\Gamma\tau)$. T_{bin} is the correlator bin width, for a linear-τ strategy, it's a constant. In this paper, T_{bin} = 1.28 µs, the lag time resolution in the SPAD settings. T_{int} is the integration time, m is the bin index, $\langle n\rangle$ is the average number of photons within the bin width T_{bin} ($\langle n\rangle = IT_{\text{bin}}$, where I is the detected photon count rate). We assumed a photon count rate of 10 kcps at ρ = 30 mm, consistent with values reported in the previous study (Ref. 39 in the main article text). During the four-layer model simulations, we observed that the unnormalized field autocorrelation function at lag time zero exhibited the relationship $G_1(\rho = 35mm, \tau = 0)/G_1(\rho = 30mm, \tau = 0) \approx$ 0.33. Based on this, we estimated a photon count rate of approximately 3.3 kcps at ρ = 35 mm.

To achieve a noise level comparable to that of the measured subject-derived noise for the DL model, we set the integration time T_{int}=180 s. Gaussian noise with zero mean and the standard deviation defined by Eq. (1) is added to the clean g_2 data to simulate realistic testing conditions for the two-layer analytical fitting.

The two-layer analytical fitting was used to simultaneously extract CBFi and extracerebral BFi (EBFi) by minimizing the single penalty function defined as:

$$\chi^2 = \sum_{i=1}^{N_\tau}\left[g_2^{\text{theory}}(\tau_i, \text{CBFi}, \text{EBFi}) - g_2^{\text{simulated}}(\tau_i, D_{b\_\text{brain}}, D_{b\_scalp})\right]^2, \quad (1)$$

where N_τ is the number of τ, and τ_i is the i'th delay time. We use *fminsearchbnd* function in MATLAB to minimize the penalty function. CBFi and EBFi are the fitting parameter with

bounds ∈ [10^{-9}, 10^{-4}] mm^2/s. The optical and physiological parameters were assumed to be known before fitting, β was assumed to be known as 0.5.

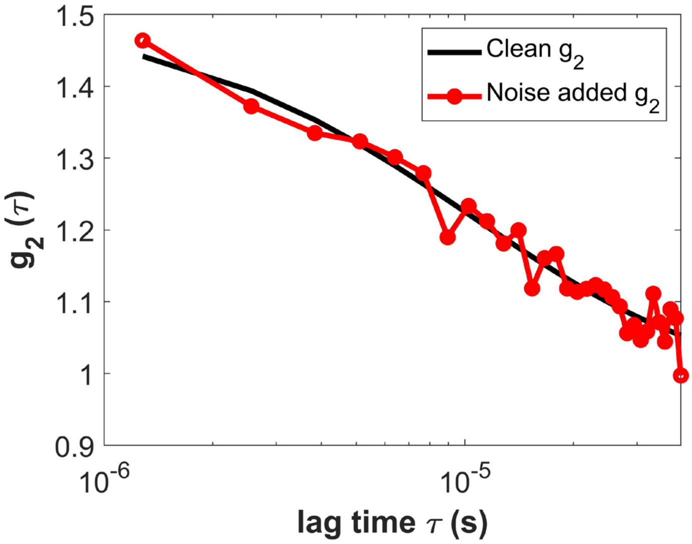

Fig. S1 An example of clean and noise-added g_2 for the two-layer analytical fitting.

2. Training loss curve

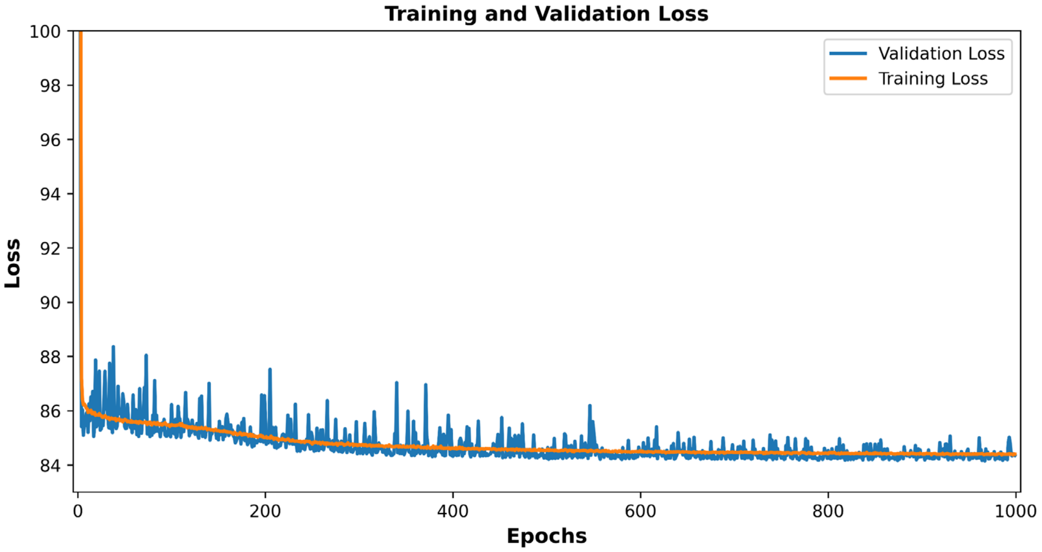

Fig. S2 MSE training and validation loss curves over 1000 epochs. The original simulated CBFi (label) was scaled by 10^6 to prevent slow training convergence, the training and validation losses converged to ~84.35 (in the scaled units). The total training time for 1000 epochs was 9.4 hours.

3. Equivalence of single-exponential fitting using original and simplified decay functions

As described in Section 2.3, we adopted a simplified function for single-exponential fitting. To validate its equivalence to the standard decay function, we compared the original form:

$$g_2(\tau) = 1 + \beta e^{-2\tau/\tau_c}, \quad (3)$$

with the simplified version:

$$g_2(\tau) = a + be^{-c\tau}. \quad (4)$$

In Eq. (3), τ_c denotes the decorrelation time. In Eq. (4), a, b, c are fitting parameters, and c is used to calculate the relative decorrelation speed (rCBFi). To compare the two formulations, we used the MC simulation-based test dataset described in Section 2.5, including 500 clean samples. For each sample, we generated 10 noise-added ACFs, yielding a total of 5,000 unscaled ACFs. These were fitted using both Eq. (3) and Eq. (4), and the estimated rCBFi values were compared using Pearson's correlation and Bland–Altman agreement analysis.

Figure S3(a) shows a representative recovered rCBFi waveform. The results demonstrate that both equations produce nearly identical rCBFi trends, although Eq. (4) yields slightly greater fluctuations. The linear regression between the two methods, shown in Fig. S3(b), indicates a strong correlation. Furthermore, the Bland–Altman plot in Fig. S3(c) confirms good agreement between the two approaches.

In summary, the simplified equation (Eq. 4) provides a valid alternative for single-exponential fitting. Given that the ATLAS DCS system is highly sensitive to environmental interference and probe motion, using a more flexible fitting form with fewer constraints may help reduce artifacts and more accurately reflect true physiological changes.

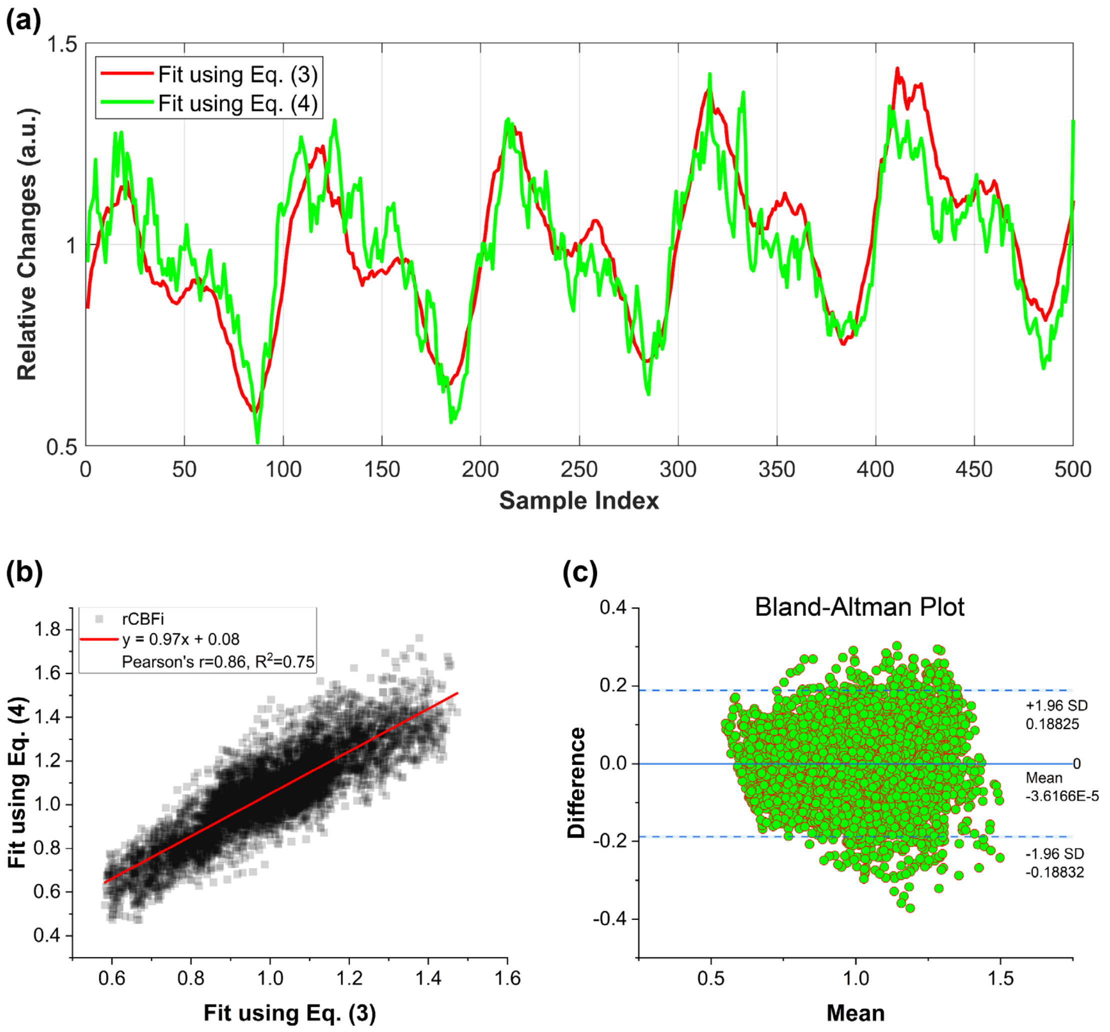

Fig. S3 Equivalence analysis of single-exponential fitting using Eq. (3) and Eq. (4) for rCBFi recovery on unscaled ACFs. (a) Representative rCBFi waveforms recovered using Eq. (3) and Eq. (4). (b) Pearson correlation analysis of rCBFi values obtained from the two fitting methods. (c) The Bland–Altman analysis showing the distribution of differences between the two methods; among the 5,000 total samples, 258 data points lie outside the limits of agreement.

4. Agreement analysis of single-exponential fitting on scaled and non-scaled ACFs

To evaluate the reliability of single-exponential fitting on the simulated test dataset, we compared its performance on both scaled and non-scaled ACFs from the baseline subject test. Fig. S4(a) shows the relative decorrelation speed estimated using both methods, which exhibit nearly identical waveforms. Fig. S4(b) presents the percentage difference between the results obtained from non-scaled and scaled ACFs, with all differences confined within ±0.5%, indicating minimal impact from the scaling process.

As shown in Fig. S4(c), the fitted values from both methods align closely along the identity line (y = x), confirming strong agreement. Additionally, the Bland–Altman plot in Fig. S4(d) further supports this finding. The differences are centered around zero with a mean bias of -2.28×10^{-7} and 95% of the data falling within the limits of agreement (±1.96 SD, or approximately ±0.00113). These results demonstrate that the scaling of ACFs has a negligible effect on the outcome of single-exponential fitting, confirming its robustness under both input formats.

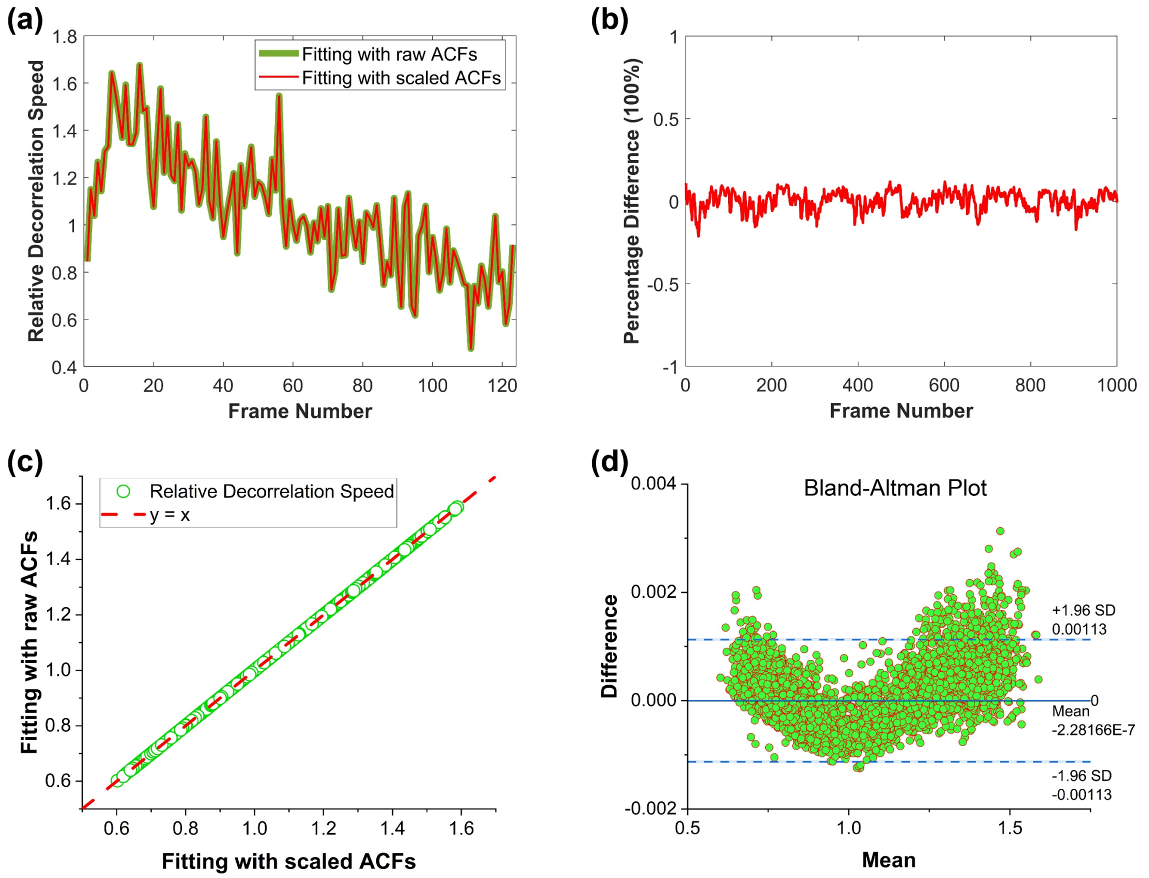

Fig. S4 Validation of the agreement between single-exponential fitting applied to scaled and non-scaled ACFs. (a) Recovered relative decorrelation speed using non-scaled and scaled ACFs during one pulse period. (b) Percentage difference between the fitted relative decorrelation speeds from the two input formats. (c) Scatter plot comparing the results from both methods against the identity line (y = x), indicating strong agreement. (d) Bland–Altman analysis showing the distribution of differences; out of 5000 total samples, 236 data points fall outside the limits of agreement.

5. Inter-subject test evaluation

To evaluate the feasibility of using our system for CBF monitoring across different subjects, we conducted additional breath-holding tests on another two healthy male participants (Subject B: 28 years old, and Subject C: 25 years old). The results, presented in Fig. S5, show that the

recovered CBF waveforms for Subjects B and C differ from one another and are less distinguishable compared to the responses observed in the primary subject. Although the system successfully captured breath-hold-induced increases in CBF for both subjects, the magnitude and pattern of the responses varied considerably (27.4% rCBFi increase for Subject B, and 5.4% increase for Subject C at the end of breath-holding).

A likely explanation for this variation is that the model was trained using a subject-specific noise profile, which may be too narrow in scope to generalize effectively to data from other individuals. We recognize this as a main limitation of the current system. In future work, we aim to improve the model's generalizability by developing more robust training dataset generation strategies and incorporating a more comprehensive, subject-independent noise model.

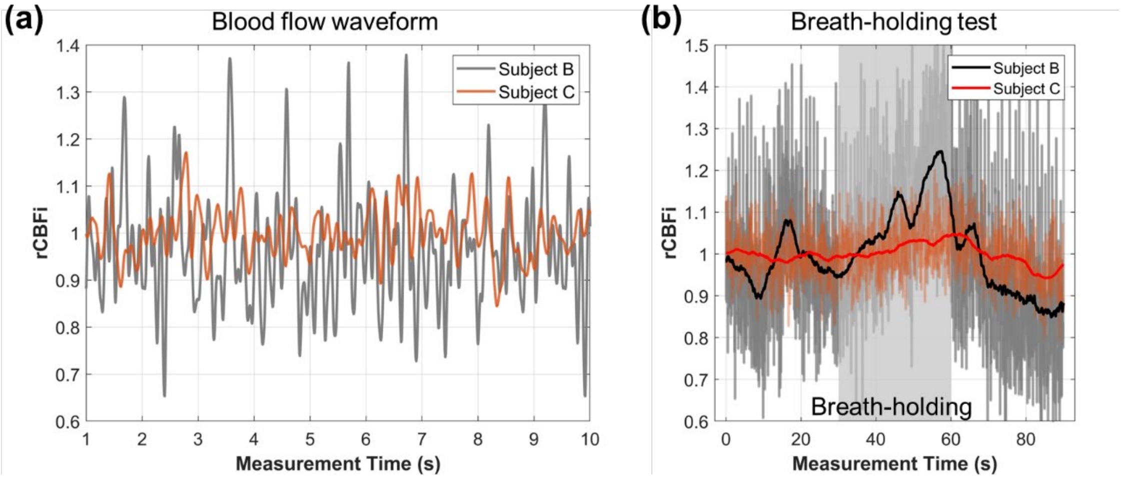

Fig. S5 Inter-subject test results. (a) Blood waveform of the first 10 seconds tested on Subject B (28-year male adult) and C (25-year male adult). (b) Breath-holding test on Subject B and C.
